# Nlrp3 inflammasome activation and Gasdermin D-driven pyroptosis are immunopathogenic upon gastrointestinal norovirus infection

**DOI:** 10.1371/journal.ppat.1007709

**Published:** 2019-04-24

**Authors:** Hanne Dubois, Frederic Sorgeloos, Soroush T. Sarvestani, Liesbet Martens, Yvan Saeys, Jason M. Mackenzie, Mohamed Lamkanfi, Geert van Loo, Ian Goodfellow, Andy Wullaert

**Affiliations:** 1 Department of Internal Medicine and Pediatrics, Ghent University, Ghent, Belgium; 2 VIB-UGent Center for Inflammation Research, VIB, Ghent, Belgium; 3 Division of Virology, Department of Pathology, University of Cambridge, Addenbrooke’s Hospital, Cambridge, United Kingdom; 4 Department of Microbiology and Immunology, University of Melbourne at the Peter Doherty Institute for Infection and Immunity, Melbourne, VIC, Australia; 5 Department of Applied Mathematics, Computer Science and Statistics, Ghent University, Ghent, Belgium; 6 Janssen Immunosciences, World Without Disease Accelerator, Janssen Pharmaceutica, Pharmaceutical Companies of Johnson & Johnson, Beerse, Belgium; 7 Department of Biomedical Molecular Biology, Ghent University, Ghent, Belgium; 8 Ghent Gut Inflammation Group (GGIG), Ghent University, Ghent, Belgium; Washington University in Saint Louis, UNITED STATES

## Abstract

Norovirus infection is the leading cause of food-borne gastroenteritis worldwide, being responsible for over 200,000 deaths annually. Studies with murine norovirus (MNV) showed that protective STAT1 signaling controls viral replication and pathogenesis, but the immune mechanisms that noroviruses exploit to induce pathology are elusive. Here, we show that gastrointestinal MNV infection leads to widespread IL-1β maturation in MNV-susceptible STAT1-deficient mice. MNV activates the canonical Nlrp3 inflammasome in macrophages, leading to maturation of IL-1β and to Gasdermin D (GSDMD)-dependent pyroptosis. STAT1-deficient macrophages displayed increased MAVS-mediated expression of pro-IL-1β, facilitating elevated Nlrp3-dependent release of mature IL-1β upon MNV infection. Accordingly, MNV-infected Stat1^-/-^ mice showed Nlrp3-dependent maturation of IL-1β as well as Nlrp3-dependent pyroptosis as assessed by *in vivo* cleavage of GSDMD to its active N-terminal fragment. While MNV-induced diarrheic responses were not affected, Stat1^-/-^ mice additionally lacking either Nlrp3 or GSDMD displayed lower levels of the fecal inflammatory marker Lipocalin-2 as well as delayed lethality after gastrointestinal MNV infection. Together, these results uncover new insights into the mechanisms of norovirus-induced inflammation and cell death, thereby revealing Nlrp3 inflammasome activation and ensuing GSDMD-driven pyroptosis as contributors to MNV-induced immunopathology in susceptible STAT1-deficient mice.

## Author summary

Gastrointestinal norovirus infections form a serious socio-economic worldwide problem, with about 684 million cases annually worldwide and mortality occurring both in underdeveloped countries and in immunocompromised patients. Despite the urgent global need, no specific treatments are available for norovirus induced gastroenteritis, partly because innate immune responses upon gastrointestinal norovirus infection are largely unresolved. Using the murine norovirus (MNV) model we showed that MNV infection in macrophages leads to a lytic form of cell death termed pyroptosis as well as to the maturation and release of the pro-inflammatory cytokine IL-1β. Maturation of IL-1β was observed also in vivo, after gastrointestinal infection of MNV-susceptible Stat1 knockout mice. We found that these innate immune responses upon MNV infection crucially depended on activation of the Nlrp3 inflammasome leading to Gasdermin D-driven pyroptosis, and inactivating this signaling pathway delayed lethality of MNV-susceptible STAT1 knockout mice after gastrointestinal MNV infection. We thus identified Nlrp3 inflammasome activation and ensuing pyroptosis as a signaling pathway contributing to norovirus-induced immunopathology. Taken together, this study resulted in a more detailed understanding of MNV-induced inflammatory and cell death pathways and provided insights into how gastrointestinal viruses induce immunopathology.

## Introduction

Human norovirus infection is the most common cause of food-borne gastroenteritis worldwide, responsible for an estimated 684 million cases per year [1]. While norovirus gastroenteritis is self-limiting in immunocompetent individuals, it can evolve to a seriously debilitating and even life-threatening infection in conditions of genetic or acquired immunosuppression [2]. Moreover, norovirus infections also account for more than 200,000 deaths annually, mostly affecting children below five years of age in developing countries [1]. Despite this significant global socio-economic burden, the cellular processes that are induced upon gastrointestinal norovirus infection are poorly understood.

Norovirus challenge studies in human volunteers showed that viral shedding lasts long after the acute vomiting and diarrhea symptoms have subsided, and also revealed that in some individuals the viral burden peaks after clinical symptoms had been resolved [3]. This poor correlation between viral titers and gastrointestinal manifestations suggests that in addition to norovirus replication, some of the host innate responses to infection may contribute to provoking pathology. Studies using the murine norovirus (MNV) model showed that Interferon (IFN)-induced STAT1-dependent responses are required for controlling viral replication and associated pathogenesis [4–6], but the deleterious innate immune mediators that contribute to norovirus-induced intestinal and systemic inflammation remain to be elucidated.

Several studies identified inflammasome activation as a crucial innate immune mechanism that protects the host from a wide variety of viral infections [7, 8]. Inflammasomes are cytosolic multi-protein complexes in which caspase-1 and -11 proteolytic activities exert important functions in innate immunity by mediating maturation of the pro-inflammatory cytokines Interleukin (IL)-1β and IL-18, as well as by initiating a lytic form of cell death termed pyroptosis through cleaving Gasdermin D (GSDMD) [9]. For instance, inflammasome activation was shown to preserve respiratory function and to prevent lethality upon Influenza infection [10–12]. Similar host protective roles for inflammasomes were reported in encephalitis caused by West Nile virus as well as in gastroenteritis induced by rotavirus [13–15]. However, the role of inflammasomes in norovirus-induced immunopathology and norovirus pathogenesis remains to be elucidated.

Here, we show that inflammasome activation has a detrimental rather than a beneficial function during gastrointestinal norovirus infection. We show that MNV activates the canonical Nlrp3 inflammasome leading to IL-1β secretion as well as GSDMD-dependent pyroptosis in macrophages. In addition, we show that these Nlrp3- and GSDMD-mediated responses do not contribute to the diarrheic manifestations of gastroenteritis but do promote MNV-induced intestinal inflammation and lethality in STAT1-deficient mice. These data show that Nlrp3 inflammasome signaling and GSDMD-driven pyroptosis contribute to immunopathology in the setting of gastrointestinal norovirus infection.

## Results

### Stat1^-/-^ mice display increased production and proteolytic maturation of IL-1β upon gastrointestinal MNV infection

As an *in vivo* model for norovirus-induced gastroenteritis with concomitant systemic inflammatory responses, we infected Stat1^-/-^ mice via oral gavage with the non-persistent MNV-1 CW1 strain [16] (hereafter referred to as MNV), which was lethal for infected Stat1^-/-^ mice but not for their Stat1^+/-^ littermates (S1A Fig). In order to identify putative innate immune mediators contributing to MNV-induced intestinal inflammation and lethality in Stat1^-/-^ mice, we first characterized *in vivo* MNV replication kinetics. To this end, cohorts of Stat1^-/-^ and Stat1^+/-^ littermate mice were orally infected with MNV and tissues were harvested at one, two or three days post-infection. Quantification of viral RNA levels showed that Stat1^-/-^ mice displayed overall significantly higher viral burdens when compared with Stat1^+/-^ littermates over the entire gastrointestinal tract, in Peyer’s patches (PP) and mesenteric lymph nodes (MLN), as well as in the spleen and liver only at 3 days post-infection (S1B–S1J Fig). Analysis of IFNβ expression levels at a time point when systemic viral spread had occurred, indicated that MNV infection of Stat1^-/-^ mice caused robust anti-viral type I IFN responses in all of these tissues examined, with the exception of the distal colon (S2 Fig). MNV genomes detected in the distal colon may therefore derive from non-replicating viral particles, or alternatively MNV-induced IFN responses in the distal colon may occur with different kinetics or may be less potent compared to other parts of the gastrointestinal tract. Nevertheless, the above analyses showed that Stat1^-/-^ mice displayed intestinal, as well as systemic dissemination of MNV at three days post-infection, which was associated with anti-viral responses within the corresponding tissues.

We next investigated how these viral replication kinetics correlated with MNV-induced diarrhea and intestinal inflammation. Consistent with the peak of viral titers in these mice, we observed that gastrointestinal MNV infection caused diarrhea only in Stat1^-/-^ mice at three days post-infection (S3A Fig). Gastrointestinal MNV infection is known to provoke only subtle inflammation in the intestine, even in highly susceptible Stat1^-/-^ mice. For instance, a study performing detailed histological examinations could not reveal increased numbers of inflammatory cells in the intestinal lamina propria of MNV-infected Stat1^-/-^ mice [17]. Therefore, to monitor intestinal inflammation in MNV-infected mice non-invasively over time in a sensitive manner we measured fecal Lipocalin-2 (Lcn-2) levels that were shown to correlate closely with varying degrees of inflammation restricted to the intestine [18]. While fecal Lcn-2 levels did not increase in MNV-infected Stat1^+/-^ mice, Stat1^-/-^ littermates displayed a significant elevation of fecal Lcn-2 levels at two and three days after oral MNV infection (S3B Fig). This observation validated fecal Lcn-2 as a sensitive marker of MNV-induced intestinal inflammation, and indicated that the kinetics of intestinal pathology correlated with previously observed viral replication and anti-viral responses. Therefore, three days post-infection was selected as an appropriate time point for screening innate immune responses potentially associated with MNV-induced intestinal and systemic pathology.

**Fig 1:**
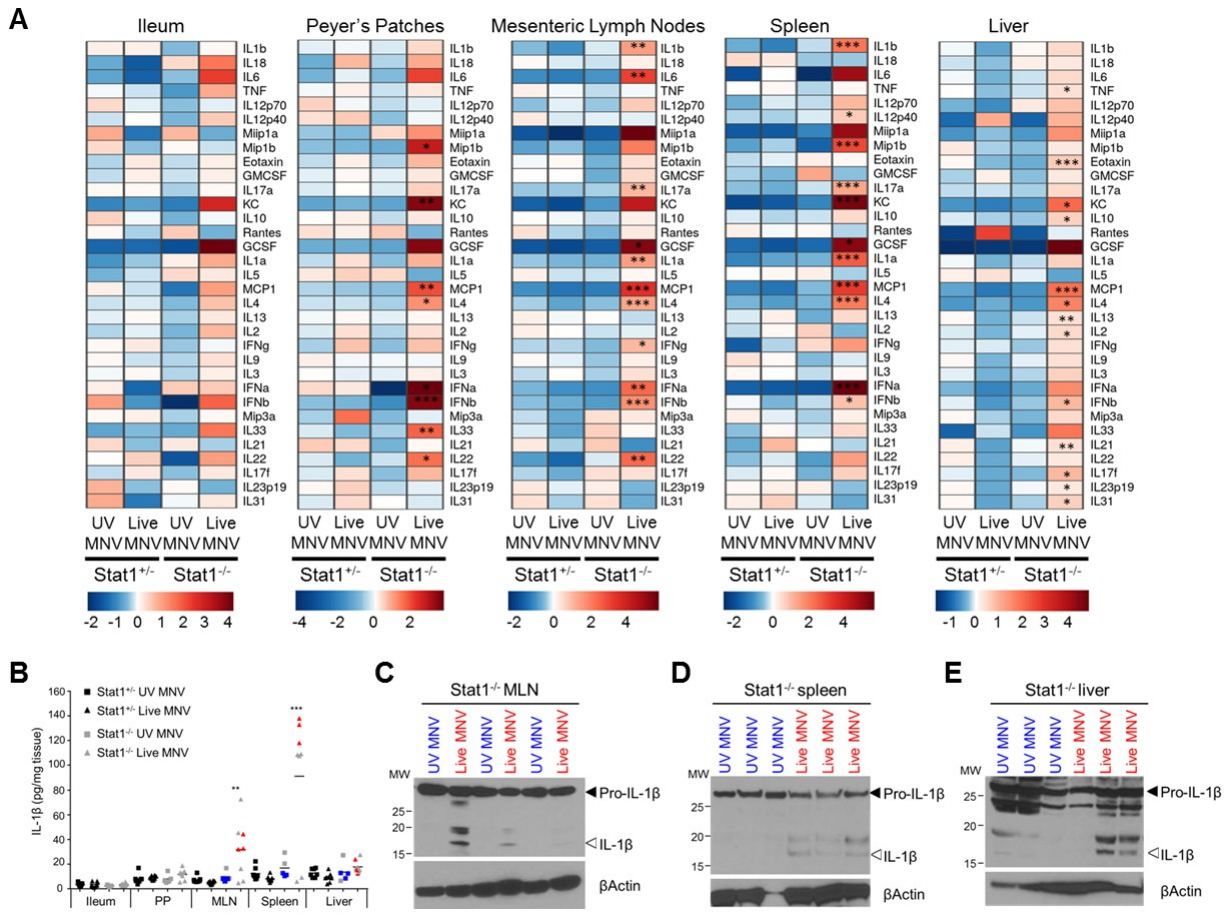
Stat1^-/-^ mice display increased production and proteolytic maturation of IL-1β upon gastrointestinal MNV infection. Age- and sex-matched Stat1^+/-^ and Stat1^-/-^ littermates were infected with 10^/^ PFU of UV-inactivated (UV) or live MNV via oral gavage. At 3 days post-infection ileum, Peyer`s patches (PP), mesenteric lymph nodes (MLN), spleen and liver lysates were prepared for Multiplex ELISA (A, B) and Western Blotting analyses (C-E). (A) Heat maps showing relative expression of chemokines and cytokines as measured by Multiplex ELISA in the indicated tissues of UV- or Live MNV infected Stat1^+/-^ and Stat1^-/-^mice. Data shown are the means of Stat1^+/-^UV-MNV mice (n = 7), Stat1^+/-^Live-MNV mice (n = 8), Stat1^-/-^UV-MNV mice (n = 5) and Stat1^-/-^Live-MNV mice (n = 8), normalized per mean log2 expression. (B) Raw data from heat map analysis showing IL-1β levels in indicated tissues. Samples indicated in blue and red were analyzed by (C-E) Western blotting for proteolytic maturation of IL-1β in (C) MLN, (D) spleen and (E) liver. Statistics: Log-linear regression analysis, p values in (A, B) indicate association with Stat1^-/-^Live-MNV set-up, with *p<0.05; **p<0.01; ***p<0.001.

For this purpose, we quantified the expression levels of a panel of inflammatory chemokines and cytokines at selected MNV replication sites in Stat1^-/-^ and Stat1^+/-^ littermates that had been infected for three days with either UV-inactivated or live MNV. Statistical log-linear regression analysis identified several chemokines and cytokines that were specifically associated with MNV-induced pathology, as defined by statistically significant higher expression levels in Stat1^-/-^ mice infected with live MNV relative to Stat1^-/-^ mice challenged with UV-inactivated MNV, as well as relative to live MNV-infected Stat1^+/-^ littermate controls (Fig 1A, S1 Table). The various inflammatory cytokines expressed at higher levels in live MNV-infected Stat1^-/-^ mice indicated a complex immune response raised to MNV in these mice. For instance, live MNV-infected Stat1^-/-^ mice displayed increased levels of IFNγ, IL-4 and IL-17A in MLN, suggestive of ongoing ILC1/Th1, ILC2/Th2 as well as ILC3/Th17 innate and/or adaptive lymphocyte responses (Fig 1A, S1 Table). Amongst the unambiguously innate immune mediators associated with severity of MNV infection in Stat1^-/-^ mice, the cell death-associated alarmin IL-1α and the inflammasome-dependent cytokine IL-1β were prominent in both MLN and the spleen (Fig 1A and 1B). Because IL-1β is produced as a cytosolic precursor protein that needs maturation by inflammasomes for exerting biological activity, we performed additional Western blotting analyses. Consistent with inflammasome activation occurring in MNV-infected mice, these analyses showed *in vivo* maturation of pro-IL-1β to its active IL-1β form in live MNV-infected Stat1^-/-^ MLN and spleen (Fig 1C and 1D). In addition, although overall IL-1β levels in liver were not significantly increased (Fig 1A and 1B), liver tissue of a subset of live MNV-infected Stat1^-/-^ mice contained detectable levels of mature IL-1β (Fig 1E). Together, these results showed that Stat1^-/-^ mice produced mature IL-1β three days after gastrointestinal MNV infection. In line with our observations showing that MNV-infected Stat1^-/-^ mice displayed detectable intestinal inflammation as well as systemic viral spread only at three days post-infection, we could not detect increased IL-1β levels in MNV-infected Stat1^-/-^ mice at earlier time points post-infection in any tissue examined (S4 Fig). These correlating cytokine, intestinal inflammation and systemic viral dissemination kinetics suggested that IL-1β could contribute to local as well as systemic MNV-induced pathologies, prompting us to further investigate the mechanisms by which MNV induces IL-1β maturation and release.

### MNV induces secretion of mature IL-1β by activating the caspase-1- and ASC-dependent Nlrp3 inflammasome

**Fig 2:**
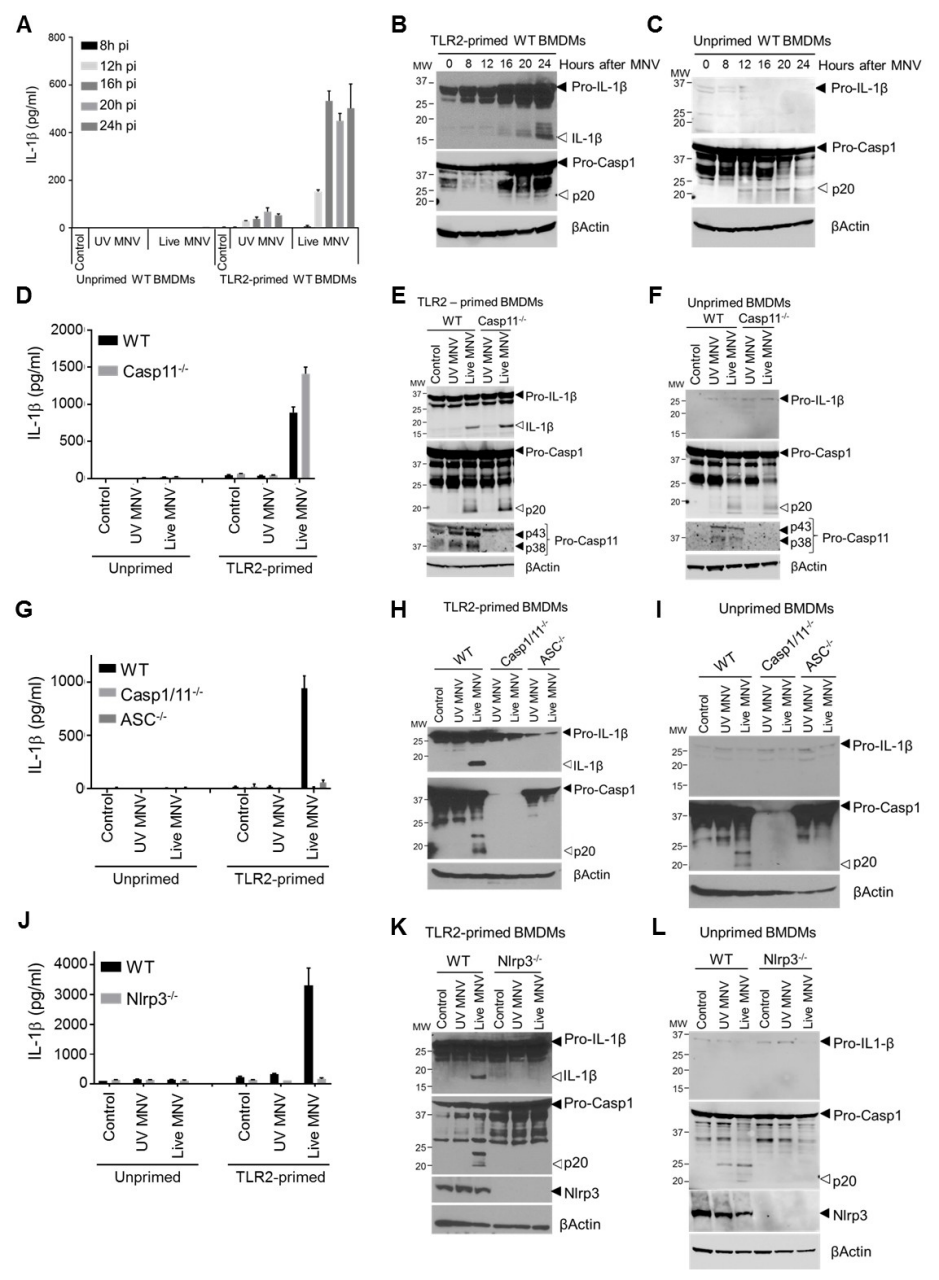
MNV induces secretion of mature IL-1β by activating the caspase-1- and ASC-dependent Nlrp3 inflammasome. Bone-marrow-derived macrophages (BMDMs) from (A-C) WT mice; (D-F) WT and Caspase-11^-/-^ mice; (G-I) WT, Caspase-1/11^-/-^ and ASC^-/-^ mice; or (J-L) WT and Nlrp3^-/-^ mice were left untreated (unprimed) or were TLR2-primed after which they were mock infected (control) or infected with either UV-inactivated or live MNV, both at a MOI 5. Cell culture supernatants collected after indicated time periods (A) or after 24h (D, G, J) were analyzed for secreted IL-1β by ELISA; and (B, C, E, F, H, I, K, L) cell lysates prepared 24h post-infection was immunoblotted for IL-1β and caspase-1 maturation. Data shown in (A, D, G, J) are the means ± SD of triplicate wells from a representative experiment out of at least two independent experiments. Data shown in (B, C, E, F, H, I, K, L) are representative for at least two independent experiments.

Inflammasomes are cytosolic multiprotein complexes that engage caspase-1 and/or -11 to exert both inflammatory and cytotoxic functions in response to infectious agents [7]. Since myeloid cells are major targets during MNV infection [16, 19, 20], we next turned to primary bone marrow-derived macrophages (BMDMs) as a controlled *ex vivo* setting to dissect the mechanisms underlying inflammasome responses to MNV infection. MNV-infected macrophages that were primed with the TLR2 agonist Pam3CSK4 secreted IL-1β into the culture supernatant starting 12–16 hours post-infection (Fig 2A). This IL-1β secretion was associated with proteolytic maturation of pro-IL-1β and caspase-1 (Fig 2B), confirming MNV-induced inflammasome activation in these cells. In contrast, unprimed MNV-infected macrophages failed to secrete IL-1β, consistent with the need for prior transcriptional upregulation of pro-IL-1β (Fig 2A). Nevertheless, these unprimed WT macrophages displayed caspase-1 processing starting 12 hours after MNV infection (Fig 2C), demonstrating that MNV itself has the capacity to trigger inflammasome activation even in the absence of sufficient amounts of pro-IL-1β that would contribute to inflammatory responses.

We next set out to dissect the composition of the MNV-activated inflammasome. Experiments in caspase-11-deficient primary macrophages showed that caspase-11 did not contribute to MNV-induced caspase-1 activation and that the latter was sufficient for mediating IL-1β maturation and secretion upon MNV infection (Fig 2D–2F). Next, MNV infections in primary macrophages lacking both caspase-1 and -11, or lacking the adaptor protein ASC demonstrated that MNV induced maturation and secretion of IL-1β in an ASC- and caspase-1-dependent manner (Fig 2G–2I). While MNV infections in Aim2-, Nlrc4- and Nlrp6-deficient primary macrophages showed that these Pattern Recognition Receptors were dispensable for MNV-induced inflammasome activation (S5A–S5D Fig), MNV did not provoke caspase-1 processing or downstream IL-1β maturation and secretion upon infecting primary or immortalized Nlrp3^-/-^ macrophages (Fig 2J–2L, S5A–S5E Fig). Importantly, despite abrogating MNV-induced inflammasome responses, the absence of Nlrp3 did not impair MNV replication rates or MNV-induced type I IFN responses in macrophages (S6A and S6B Fig), showing that MNV-induced inflammasome activation does not interfere with the protective IFN responses that are triggered upon MNV dsRNA recognition, at least *in vitro*. Together, the above observations established activation of the canonical Nlrp3 inflammasome as an innate immune pathway responsible for IL-1β secretion from MNV-infected macrophages.

### Nlrp3-dependent GSDMD cleavage provokes pyroptosis in MNV-infected macrophages

**Fig 3:**
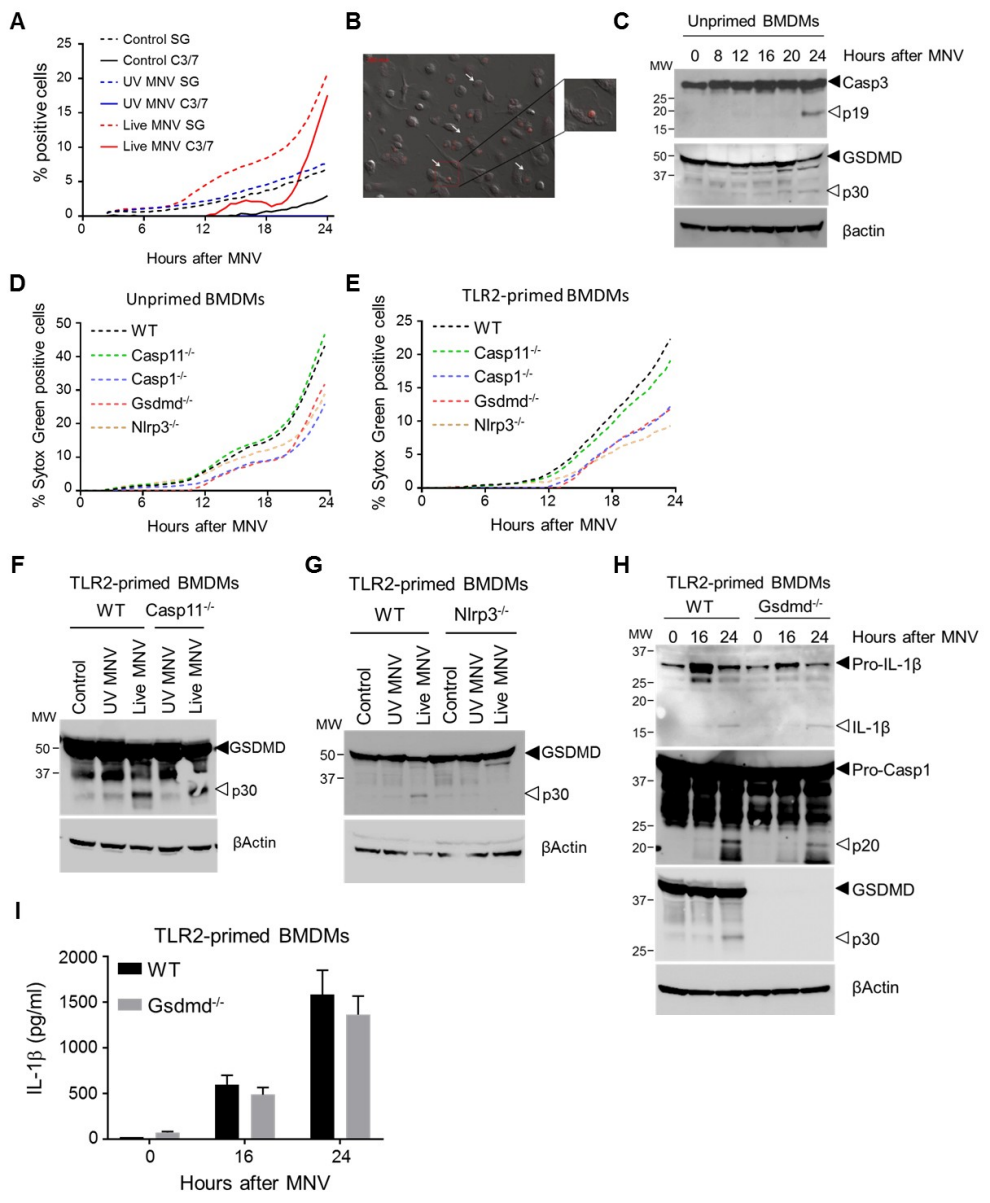
Nlrp3-dependent GSDMD cleavage mediates pyroptosis in MNV-infected macrophages. (A-C) Unprimed WT bone-marrow-derived macrophages (BMDMs) were infected with either UV-inactivated or live MNV at a MOI 5. (A) Sytox Green (SG) uptake and Caspase-3/-7 (C3/7) activity were assessed for 24 hours by IncuCyte real-time monitoring every 30 minutes; (B) confocal microscopic picture of Propidium Iodide uptake and cell morphology at 13 hours post-infection. White arrows indicate cells displaying swollen necrotic morphology; (C) cell lysates were prepared at indicated time points post-infection and were immunoblotted for GSDMD and caspase-3 processing. (D-I) BMDMs from WT and indicated KO mice were (D) left untreated (unprimed) or were (E-I) TLR2-primed, after which they were infected with either UV-inactivated or live MNV at a MOI 5. (D, E) Sytox Green (SG) uptake was assessed for 24 hours by IncuCyte real-time monitoring every 30 minutes. (F-H) Cell lysates were prepared at 24 hours post-infection and were immunoblotted for (F-G) GSDMD processing along with (H) IL-1β and caspase-1 maturation. (I) Cell culture supernatants collected at indicated time points post-infection were analyzed for secreted IL-1β. Data shown in (A, D-E) are the means of duplicate wells from a representative experiment out of two independent experiments; data shown in (B-C, F-H) are representative for at least two independent experiments; data shown in (I) are the means ± SD of triplicate wells from a representative experiment out of two independent experiments.

We next investigated the cellular mechanisms by which MNV-induced inflammasome activation mediated secretion of IL-1β. Several MNV infection studies using RAW264.7 macrophages showed that MNV provokes apoptotic cell death [21–24]. However, analyzing the kinetics of apoptotic signaling and cellular membrane permeability by simultaneous real-time monitoring of cleavage of a fluorogenic caspase-3/7 substrate and of cell-impermeable Sytox Green (SG) uptake, respectively, showed that MNV-infected primary macrophages displayed SG uptake due to membrane permeability starting from 12 hours post-infection, while caspase-3/7 activity was detected only at later stages of MNV infection (Fig 3A). Consistent with this observation arguing against an exclusive role for apoptosis in MNV-induced cell death, confocal live cell imaging of MNV-infected primary macrophages revealed cells undergoing an increase in membrane permeability followed by cellular swelling, characteristic of a necrotic cell death mode (Fig 3B, S1 Movie). Importantly, the above real-time and imaging analyses detected MNV-induced membrane permeability and cellular swelling from 12 hours post-infection, similar as observed for caspase-1 maturation and IL-1β secretion (see Fig 2A–2C). These concordant kinetics suggested a functional link between inflammasome activation and loss of membrane integrity. Inflammasomes are known to provoke a lytic cell death mode termed pyroptosis. The biochemical hallmark of inflammasome-induced pyroptosis is the proteolytic processing of Gasdermin D (GSDMD), resulting in its N-terminal p30 fragment capable of generating pores in the cellular membrane that eventually lyse the cell due to osmotic pressure build-up [25–30]. We therefore performed Western blotting analyses showing that MNV-infected BMDMs displayed GSDMD processing to its p30 fragment starting at 12 hours post-infection (Fig 3C), in line with previously observed kinetics of inflammasome activation. In contrast, proteolytic activation of caspase-3 indicative of apoptotic cell death was detected only at 24 hours after MNV infection (Fig 3C). Together, the above real-time imaging and biochemical observations suggested that apoptosis is a late-stage event after infecting primary macrophages with MNV, whereas a population of MNV-infected macrophages undergo an earlier lytic cell death associated with GSDMD processing, indicative of pyroptosis.

After observing MNV-induced GSDMD processing concordant with the appearance of lytic cell death, we next sought to validate the functional role of GSDMD in MNV-induced cell death and IL-1β release. Real-time SG uptake analyses showed that both unprimed and TLR2-primed primary Gsdmd^-/-^ macrophages displayed a delayed membrane permeability increase upon MNV infection (Fig 3D–3E). Furthermore, in accordance with our above observations identifying Nlrp3 and caspase-1 but not caspase-11 as inflammasome components engaged by MNV (see Fig 2D–2L), Nlrp3^-/-^ and Casp1^-/-^ macrophages showed a delay in MNV-induced membrane permeability similar to Gsdmd^-/-^ cells, while Casp11^-/-^ macrophages responded like WT cells (Fig 3D–3E). Further biochemical evidence for inflammasome-dependent pyroptosis in MNV-infected macrophages was obtained by Western blotting analyses showing that MNV-induced generation of the GSDMD p30 fragment did not require caspase-11 (Fig 3F) but was dependent on the presence of Nlrp3 (Fig 3G), consistent with the opposing roles of these proteins in MNV-induced inflammasome activation.

We next evaluated the role of GSDMD-mediated pyroptosis in MNV-induced release of IL-1β from macrophages. While GSDMD deficiency did not affect maturation of caspase-1 or pro-IL-1β (Fig 3H)–consistent with its role downstream of inflammasome activation–also MNV-induced secretion of IL-1β into the culture supernatant was not diminished in Gsdmd^-/-^ macrophages (Fig 3I). This persisting IL-1β secretion in MNV-infected Gsdmd^-/-^ macrophages aligns with the incomplete blockade of SG uptake in these cells, and supports the existence of additional mechanisms contributing to cellular permeability during MNV infection. As IL-1β secretion triggered by canonical inflammasomes indeed was reported to involve also GSDMD-independent mechanisms [25], we investigated whether necroptosis contributed to MNV-induced IL-1β release. Necroptosis is executed by the pseudokinase MLKL, which upon phosphorylation creates nanopores in the plasma membrane leading to cellular swelling and lysis [31, 32]. This MLKL-induced membrane damage was suggested as a trigger for Nlrp3 inflammasome activation [33, 34], raising the possibility that upon MNV infection, MLKL could act upstream to activate the Nlrp3 inflammasome as well as downstream to help release mature IL-1β through necroptosis. However, real-time SG uptake measurements did not reveal delayed membrane permeability in MNV-infected Mlkl^-/-^ macrophages (S7A and S7B Fig). In accordance, the kinetics of MNV-induced inflammasome activation and release of mature IL-1β were unchanged in Mlkl^-/-^ macrophages (S7C–S7E Fig). Together, these results showed that MLKL was not required for MNV-induced inflammasome responses. In addition, these results dismissed a role for MLKL-mediated necroptosis as a GSDMD-independent lytic cell death contributing to IL-1β release from MNV-infected cells, leaving apoptotic cells as the remaining potential source of IL-1β secretion. Interestingly, recent studies showed that GSDMD-deficient macrophages could relay inflammasome signaling into the execution of apoptotic cell death [35, 36]. In support of this idea, real time monitoring of a fluorogenic caspase-3/7 substrate in MNV-infected BMDMs showed increased apoptotic activity in the absence of GSDMD (S8A Fig). Moreover, Gsdmd^-/-^ BMDMs also showed earlier and stronger caspase-3 cleavage upon MNV infection compared to WT BMDMs (S8B Fig). Together, these observations showing increased apoptotic signaling in Gsdmd^-/-^ macrophages suggest that the residual IL-1β release that is detected in the absence of GSDMD-driven pyroptosis might derive from apoptotic cell bodies that lyse through secondary necrosis.

### STAT1 controls MNV-induced inflammasome-mediated IL-1β responses by restricting MAVS-mediated pro-IL-1β production

**Fig 4:**
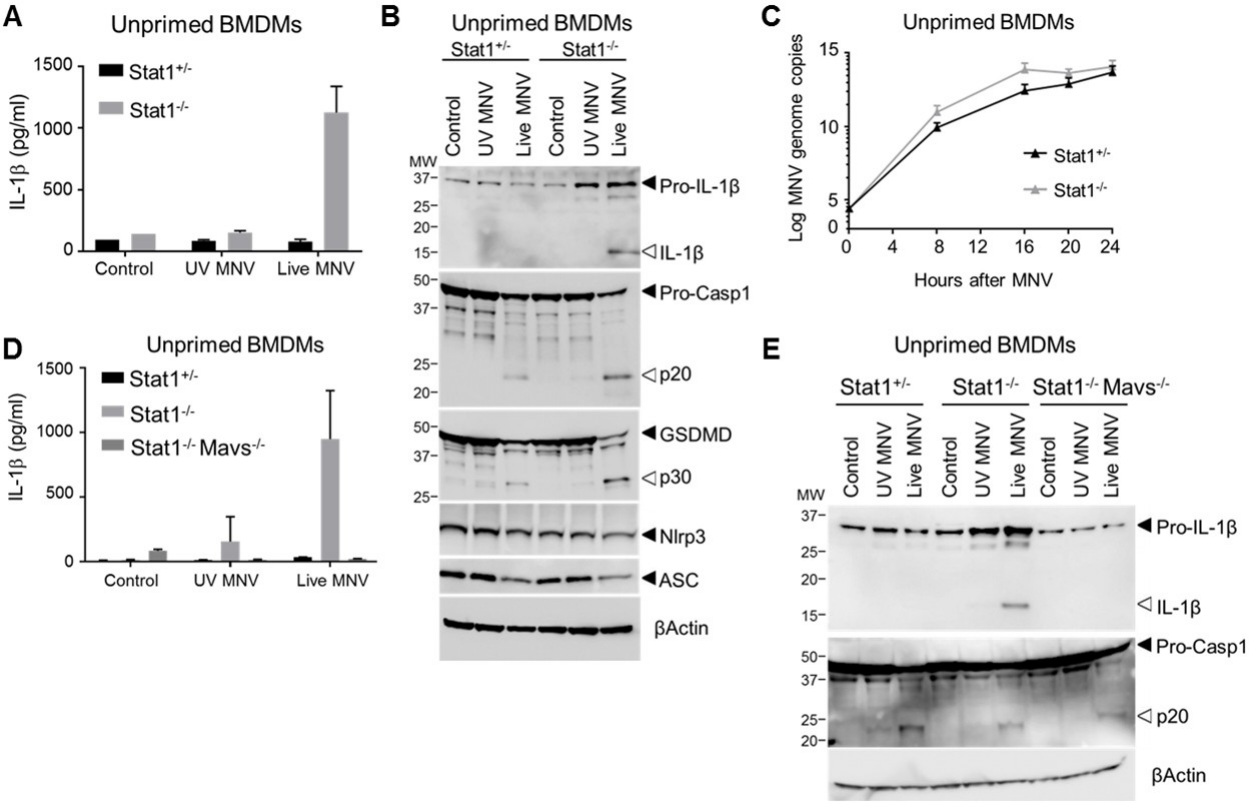
STAT1 suppresses the secretion of mature IL-1β from unprimed MNV-infected macrophages through inhibiting MAVS-mediated pro-IL-1β induction. (A-C) Unprimed bone-marrow-derived macrophages (BMDM) from Stat1^+/-^ and Stat1^-/-^ littermates were mock infected (control) or infected with either UV-inactivated or live MNV, both at a MOI 5. After 24 hours (A) culture supernatants were analyzed for secreted IL-1β by ELISA; (B) cell lysates were immunoblotted for IL-1β, caspase-1, and GSDMD processing; and (C) at indicated time points MNV genome copy numbers in BMDMs were determined. (D-E) Unprimed bone-marrow-derived macrophages (BMDM) from Stat1^+/-^, Stat1^-/-^ and Stat1^-/-^Mavs^-/-^ mice were mock infected (control) or infected with either UV-inactivated or live MNV, both at a MOI 5. After 24 hours (D) supernatant was analyzed for secreted IL-1β levels by ELISA; and (E) cell lysates were immunoblotted for IL-1β and caspase-1 processing. Data shown in (A, D) are the means ± SD of triplicate wells from a representative experiment out of two independent experiments. Data shown in (B, E) are representative for 2 independent experiments. Data shown in (D) represent means ± SD of BMDMs derived from three mice per genotype, analyzed in three independent experiments, each with triplicate wells.

As our above observations pointed to a dual cytokine maturation and cell death inducing role of the Nlrp3 inflammasome upon MNV infection, we next evaluated the role of Nlrp3 in MNV-susceptible Stat1^-/-^ conditions. Interestingly, we noted pronounced IL-1β secretion from Stat1^-/-^ macrophages without the need for prior TLR2 priming (Fig 4A). Indeed, while caspase-1 activation and downstream GSDMD p30 generation were evident in both unprimed Stat1^+/-^ and unprimed Stat1^-/-^ macrophages, STAT1 deficiency was additionally associated with pro-IL-1β processing leading to secretion of its mature form (Fig 4A and 4B). The observed effect of STAT1 deficiency allowing IL-1β maturation in unprimed macrophages was specific for MNV-induced Nlrp3 inflammasome activation, as unprimed Stat1^-/-^ macrophages did not display IL-1β maturation upon triggering the Nlrp3 inflammasome with ATP, Nigericin or MSU (S9 Fig). Closer Western blotting examination of unprimed MNV-infected Stat1^-/-^ macrophages revealed that STAT1 deficiency was associated with enhanced expression of pro-IL-1β but not of the Nlrp3 or ASC inflammasome components (Fig 4B). Since MNV-infected unprimed Stat1^-/-^ macrophages displayed faster MNV replication rates compared to Stat1^+/-^ cells (Fig 4C), more pronounced pro-IL-1β expression could be caused by enhanced dsRNA-induced NF-κB signaling triggered by increased viral RNA. Therefore, because MNV-induced NF-κB responses rely on dsRNA triggering of the MDA5-MAVS pathway [37], we examined the role of MAVS in MNV-induced inflammasome responses. Deleting MAVS in otherwise WT BMDMs abrogated MNV-induced IFNβ secretion as expected (S10A Fig), but did not alter MNV-induced inflammasome responses as assessed by IL-1β secretion as well as by pro-IL-1β, caspase-1 and GSDMD processing (S10B–S10D Fig), showing that MNV-induced activation of the Nlrp3 inflammasome does not rely on MAVS signaling. Interestingly however, unlike unprimed STAT1-deficient macrophages, unprimed MAVS-deficient macrophages did not display increased pro-IL-1β expression upon MNV infection and did not alter MNV replication rates in unprimed conditions (S10D and S10E Fig). These pronounced differences between the effects of deleting either STAT1 or MAVS during MNV infection of unprimed BMDMs supported the idea that enhanced MNV-induced signaling associated with increased viral replication could explain the propensity of unprimed Stat1^-/-^ macrophages to boost pro-IL-1β expression. Indeed, abrogating MAVS-mediated signaling in Stat1^-/-^ macrophages did not only abolish MNV-induced IFNβ secretion as expected (S11 Fig), but the resulting Stat1^-/-^Mavs^-/-^ BMDMs also lost their ability to secrete IL-1β upon MNV infection in unprimed conditions (Fig 4D). Additional MAVS deletion in Stat1^-/-^ macrophages did not alter inflammasome responses in terms of caspase-1 cleavage (Fig 4E), consistent with its dispensable role in MNV-induced inflammasome activation as observed on a WT background, but it abrogated the increase in pro-IL-1β levels observed in unprimed Stat1^-/-^ BMDMs, resulting in the lack of IL-1β maturation in unprimed MNV-infected Stat1^-/-^Mavs^-/-^ BMDMs (Fig 4E). Together, our observations in MNV-infected Stat1^-/-^, Mavs^-/-^ and Stat1^-/-^Mavs^-/-^ macrophages showed that STAT1 controls MNV-induced inflammasome-mediated IL-1β responses by restricting MAVS-mediated signaling towards upregulation of pro-IL-1β levels. However, while being responsible for this MNV-induced priming of the inflammasome, MAVS signaling does not participate in the Nlrp3 activation step of MNV-induced inflammasome responses.

### Nlrp3 inflammasome activation and GSDMD-driven pyroptosis contribute to MNV-induced lethality in Stat1^-/-^ mice

**Fig 5:**
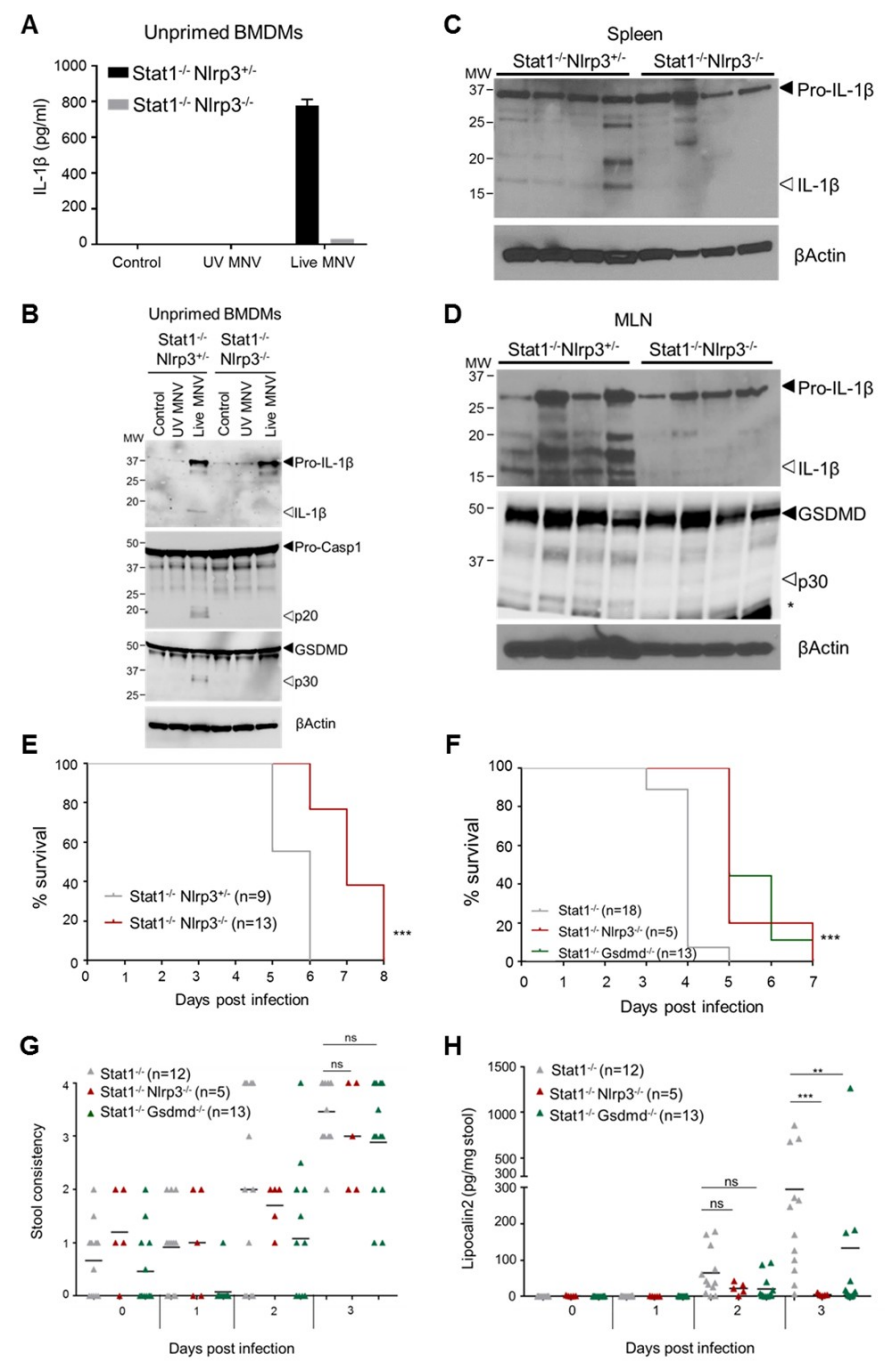
Nlrp3 as well as GSDMD contribute to MNV-induced lethality and intestinal inflammation in Stat1^-/-^ mice. (A, B) Unprimed BMDMs from Stat1^-/-^Nlrp3^+/-^ and Stat1^-/-^Nlrp3^-/-^ mice were left untreated (Control) or infected with either UV-inactivated or live MNV at MOI 5. 24 hours post-infection (A) culture supernatant was analyzed for secreted IL-1β and (B) cell lysates were immunoblotted for IL-1β, caspase-1 and GSDMD maturation. (C) Age- and sex-matched Stat1^-/-^Nlrp3^+/-^ and Stat1^-/-^Nlrp3^-/-^ littermates were infected with 10^/^ PFU MNV via oral gavage. Four days post-infection (C) spleen and (D) MLN lysates were prepared and were immunoblotted for proteolytic maturation of (C-D) IL-1β and (D) GSDMD. Each lane represents a different mouse. (E) Kaplan-Meier survival curve of Stat1^-/-^Nlrp3^+/-^ and Stat1^-/-^Nlrp3^-/-^ littermates infected with 10^6^ PFU of MNV via oral gavage. (F) Kaplan-Meier survival curve, (G) fecal consistency and (H) fecal Lipocalin-2 levels at indicated days post-infection of age- and sex-matched Stat1^-/-^, Stat1^-/-^Nlrp3^-/-^ and Stat1^-/-^Gsdmd^-/-^ mice infected with 10^6^ PFU of MNV via oral gavage. Six Stat1^-/-^ mice from (F) were unable to produce feces at three days post-infection and were therefore omitted from the analyses in (G) and (H). Data shown in (A) are the means ± SD of triplicate wells from a representative experiment out of 2 independent experiments. Data shown in (B) is representative for 2 independent experiments. Data shown in (E-H) represent graphs each combined from 2 independent experiments. Statistics (E, F) Nonparametric log-rank test; (G, H) Mann Whitney U-test; ns not significant; *p<0.05; **p<0.01; ***p<0.001.

The above *ex vivo* experiments indicated that STAT1 signaling prevents the pro-inflammatory effects of MNV-induced Nlrp3 inflammasome activation by suppressing expression of its pro-IL-1β substrate. As this could be one of the mechanisms by which STAT1 signaling controls MNV pathogenesis, we generated Stat1^-/-^Nlrp3^-/-^ mice to investigate whether Nlrp3 inflammasome-mediated IL-1β secretion contributes to the MNV-susceptible phenotype conferred by STAT1 deficiency. Whereas both MNV-infected Stat1^-/-^Nlrp3^-/-^ and Stat1^-/-^Nlrp3^+/-^ macrophages produced pro-IL-1β in the absence of TLR priming, MNV only triggered caspase-1 activation, GSDMD cleavage, pro-IL-1β maturation as well as IL-1β secretion in Stat1^-/-^Nlrp3^+/-^ cells (Fig 5A–5B). This demonstrated that Nlrp3 deficiency prevents the enhanced secretion of bio-active IL-1β from Stat1^-/-^ macrophages by blocking its maturation (Fig 5B). To evaluate whether Nlrp3 was also responsible for inflammasome responses upon MNV infection *in vivo*, Stat1^-/-^Nlrp3^-/-^ and Stat1^-/-^Nlrp3^+/-^ littermates were infected with MNV through the oral route. Western blotting analysis showed that both spleen and MLN from MNV-infected Stat1^-/-^Nlrp3^+/-^ mice displayed maturation of IL-1β, which was abrogated in MNV-infected Stat1^-/-^Nlrp3^-/-^ organs (Fig 5C–5D). Moreover, these analyses revealed that MLN from MNV-infected Stat1^-/-^Nlrp3^+/-^ mice also displayed Nlrp3-dependent GSDMD cleavage after MNV infection, indicative of pyroptosis (Fig 5D). Together, these observations demonstrated that gastrointestinal MNV infection in Stat1^-/-^ mice elicits Nlrp3-dependent IL-1β responses in MLN and spleen, as well as Nlrp3-dependent pyroptosis in MLN based on the appearance of the N-terminal p30 fragment of GSDMD as its biochemical hallmark.

We next evaluated the pathophysiological outcome of Nlrp3-mediated inflammasome responses by assessing MNV-induced lethality in Stat1^-/-^Nlrp3^-/-^ mice. Gastrointestinal MNV infections revealed that Stat1^-/-^Nlrp3^-/-^ mice survived statistically significantly longer than their Stat1^-/-^Nlrp3^+/-^ littermates (Fig 5E), clearly showing that abrogating Nlrp3-mediated IL-1β maturation has a beneficial effect in overall MNV-induced immunopathology in STAT1-deficient mice. Next, to examine whether GSDMD-driven pyroptosis takes part in the Nlrp3-mediated effects during MNV-induced immunopathology, we also generated Stat1^-/-^Gsdmd^-/-^ mice. Interestingly, Stat1^-/-^Gsdmd^-/-^ mice phenocopied the statistically significant survival advantage of Stat1^-/-^Nlrp3^-/-^ mice upon gastrointestinal MNV infection when compared to Stat1^-/-^ mice (Fig 5F). This observation indicated that also GSDMD-driven pyroptosis is crucially involved in the deleterious systemic effects of MNV contributing to lethality in Stat1^-/-^ mice. Finally, in order to evaluate whether Nlrp3 inflammasome activation and GSDMD-driven pyroptosis also exerted deleterious effects locally in the intestinal tract, we evaluated diarrhea responses and we measured fecal Lcn-2 levels in MNV-infected Stat1^-/-^Nlrp3^-/-^ and Stat1^-/-^Gsdmd^-/-^ mice. These analyses showed that gastrointestinal MNV infection provoked similar degrees of diarrhea in Stat1^-/-^Nlrp3^-/-^ and Stat1^-/-^Gsdmd^-/-^ mice when compared with Stat1^-/-^ mice (Fig 5G), indicating that inflammasome activation does not take part in MNV-induced diarrheic responses. In contrast, the induction of fecal Lcn-2 observed in MNV-infected Stat1^-/-^ mice at three days post-infection was significantly diminished in both Stat1^-/-^Nlrp3^-/-^ and Stat1^-/-^Gsdmd^-/-^ mice (Fig 5H). Taken together, while not overtly involved in the diarrhea responses that are most relevant to norovirus gastroenteritis in immunocompetent humans, the fecal Lcn-2 results show that Nlrp3 inflammasome and GSDMD pyroptosis responses do not only take part in systemic inflammatory responses that most likely underlie MNV-induced lethality, but also contribute to local MNV-induced inflammation in the intestine. Thus, our *in vivo* observations identify activation of the Nlrp3 inflammasome and ensuing GSDMD-driven pyroptosis as a critical innate immune response to MNV that–in contrast to the previously described MNV-induced signaling pathways leading to protective IFN responses [37, 38]–exerts an immunopathologic role upon gastrointestinal MNV infection in Stat1^-/-^ mice.

## Discussion

In this study, we reveal activation of the canonical Nlrp3 inflammasome leading to IL-1β secretion and GSDMD-dependent pyroptotic cell death as a detrimental innate immune response triggered by MNV. Given that several studies demonstrated protective roles for inflammasomes in various viral infections [10–15], our observation that the Nlrp3 inflammasome contributes to MNV-induced lethality in Stat1^-/-^ mice is remarkable. Interestingly however, Influenza infection studies have shown that the level of host IFN responsiveness determines the physiological outcome of inflammasome activation in viral infections. While inflammasome deficiency was detrimental during Influenza infection when compared with wild-type mice [10–12], abrogating inflammasome signaling prevented Influenza-induced lethality in Tlr7^-/-^Mavs^-/-^ mice that are impaired in mounting type I IFN responses to Influenza [39]. Our study showing that the Nlrp3 inflammasome contributes to lethality upon gastrointestinal MNV infection in IFN-unresponsive Stat1^-/-^ mice is in line with this observation in Influenza-infected mice. Moreover, our observation that STAT1 signaling prevents MAVS-mediated upregulation of pro-IL-1β suggests that enhanced expression of this inflammasome substrate may be one of the reasons why inflammasome activation is detrimental rather than protective in the absence of IFN signaling.

Although enhanced pro-IL-1β expression enables Stat1^-/-^ cells to produce more mature IL-1β upon inflammasome activation, releasing this mature IL-1β across the plasma membrane is still needed to provoke the overwhelming inflammatory responses that may contribute to lethality in Stat1^-/-^ mice. While necroptosis was suggested to perform such a role upon infection with Influenza [40, 41], MNV did not provoke MLKL-mediated membrane permeability but rather induced GSDMD-mediated pyroptosis. Although this contrasts with previous studies showing that MNV infection caused apoptotic cell death in RAW264.7 macrophages [21–24], the latter cell line is known to lack ASC expression [42]. This implies that inflammasome activation and downstream pyroptosis induction were defective in these cells. As such, apoptotic cell death in MNV-infected RAW264.7 cells is in line with our observation that primary macrophages display Casp3/7 activity at late stages upon MNV infection, and this notion supports a balance between pyroptosis and apoptosis during MNV infection in macrophages. Accordingly, we observed that MNV infection provoked elevated Casp3/7 activity and more pronounced caspase-3 cleavage in the absence of GSDMD, suggesting that Gsdmd^-/-^ cells can switch to apoptotic cell death upon MNV infection. This could explain our observation that MNV-infected GSDMD-deficient macrophages still released IL-1β, as secondary necrosis of apoptotic cell bodies may also contribute to IL-1β release in macrophage cultures. Despite this *in vitro* observation predicting that GSDMD deficiency would not be able to prevent MNV-induced inflammasome-mediated immunopathology, our *in vivo* experiments demonstrated that Stat1^-/-^Gsdmd^-/-^ mice reproduced both the survival advantage and the diminished fecal Lcn-2 levels of Stat1^-/-^Nlrp3^-/-^ mice upon gastrointestinal MNV infection. These concordant observations in Stat1^-/-^Gsdmd^-/-^ and Stat1^-/-^Nlrp3^-/-^ mice indicate that Nlrp3 inflammasome-induced pyroptosis represents the dominant cell death mode promoting MNV-induced immunopathology. While difficult to obtain experimental evidence explaining the discordant *in vitro* and *in vivo* observations, it is plausible that the IL-1β release deriving from post-apoptotic necrosis of Gsdmd^-/-^ cells *in vitro* is efficiently prevented by timely efferocytosis of apoptotic cells *in vivo*. Further supporting our observations pointing to Nlrp3-mediated pyroptosis as the physiologically relevant cell death mode during gastrointestinal MNV infection, Van Winkle *et al*. recently showed that MNV induced a lytic cell death mode in bone-marrow derived dendritic cells as well as in the BV2 microglia cell line [20]. Moreover, the ability of different MNV strains to induce this lytic cell death correlated with their persistence in the host, as lytic cell death was accompanied with myeloid cell recruitment to the MLN and the PP that provided MNV with novel target cells [20]. Although future experiments will be required to validate this hypothesis, our observations suggest that this cell death mediated process supporting systemic persistence of acute MNV infection could involve Nlrp3-induced and GSDMD-dependent pyroptosis.

In conclusion, this work contributed to a more detailed understanding of norovirus-induced inflammatory and cell death pathways *in vitro* and *in vivo*. We identify Nlrp3 inflammasome activation and downstream GSDMD-dependent pyroptosis as an MNV-induced innate immune and cell death response. We demonstrate that Nlrp3 inflammasome activation by MNV provokes both GSDMD-dependent pyroptosis as well as secretion of mature IL-1β in macrophages. We further show that these inflammasome responses contribute to lethality in MNV-susceptible Stat1^-/-^ mice. In addition, Stat1^-/-^Gsdmd^-/-^ and Stat1^-/-^Nlrp3^-/-^ mice showed diminished fecal Lcn-2 levels upon gastrointestinal MNV infection. Although the latter observations show that inflammasome responses also act locally to promote MNV-induced intestinal inflammation, diarrheic reactions upon gastrointestinal MNV infection were not decreased in Stat1^-/-^Gsdmd^-/-^ or Stat1^-/-^Nlrp3^-/-^ mice. This indicates that the mechanisms regulating intestinal inflammation and diarrhea in MNV-infected Stat1^-/-^ mice are uncoupled, and suggests that the immunopathologic role of inflammasome activation in Stat1^-/-^ mice may not be extrapolated to human norovirus gastroenteritis as occurring in immunocompetent individuals. Instead, our observations showing inflammasome responses upon systemic MNV dissemination such as occurring in Stat1^-/-^ mice suggest that Nlrp3 inflammasome activation and GSDMD-driven pyroptosis may represent an immunopathologic response that could be more relevant in life-threatening cases of human norovirus infections such as in immunocompromised individuals [2].

## Materials and methods

### Ethics statement

All animal experiments were performed according to institutionally approved protocols according to national (Belgian Laws 14/08/1986 and 22/12/2003, Belgian Royal Decree 06/04/2010) and European (EU Directives 2010/63/EU, 86/609/EEG) animal regulations. Animal protocols were reviewed and approved by the Ethical Committee Animal Experimentation VIB site Ghent—Ghent University—Faculty of Sciences (permit number LA1400091) with approval ID 2016–030. All necessary efforts were made to minimize suffering of the animals.

### Mice

Stat1^−/−^, ASC^−/−^, Casp1/11^−/−^, Casp1^−/−^, Casp11^−/−^, Nlrp3^−/−^, Nlrp6^-/-^, Nlrc4^−/−^, Aim2^−/−^, Gsdmd^−/−^, Mlkl^−/−^ and Mavs^-/-^ mice, either generated on C57BL/6 background or backcrossed at least ten generations to C57BL/6J background, were described previously [25, 43–52]. In all in vivo experiments as well as ex vivo primary cell experiments, controls were either in-house bred C57BL/6J mice (WT) or the +/+ or +/- littermates of the respective mice as indicated.

All animal experiments were performed according to approved protocols according to institutional, national and European guidelines. All mice used in this study were bred and housed in individually ventilated cages (IVC) in the Specific Pathogen Free facility at Ghent University, were sex- and age-matched and were fed autoclaved standard rodent feed (Ssniff, Soest, Germany) at libitum with free access to drinking water. In all experiments, up to 5 mice were housed per cage in a 12-h light-12-h dark cycle. Mice were assigned to experimental groups according to genotype and treatment.

### MNV infection of macrophages

Bone-marrow-derived macrophages (BMDMs) were differentiated in Iscove’s modified Dulbecco’s medium (IMDM) supplemented with 30% L929 cell conditioned medium, 10% heat-inactivated FBS, 1% Gibco non-essential amino acids and 1% penicillin/streptomycin at 37°C and 5% CO_2_. After 6 days, the differentiation medium was aspirated and the cells were scraped in cold infection medium (IMDM supplemented with 10% heat-inactivated FBS and 1% Gibco non-essential amino acids). Then, 8.5 x 10^5^ cells per well were seeded in a 12-well plate and incubated overnight at 37°C and 5% CO_2_. On day 7 the MNV infection experiments were initiated, all with the MNV-1 CW1 strain [53]. Macrophages derived from immortalized myeloid progenitors (generated by Eicke Latz, Institute of Innate Immunity, Bonn, Germany; and kindly provided by Dr. Ashley Mansell, Centre for Innate Immunity and Infectious Diseases, Clayton, Australia) were grown in DMEM (Gibco) supplemented with 10% heat-inactivated FBS and 2 mM glutamine.

For MNV infection experiments, unprimed macrophages were used, or macrophages were first primed with TLR agonists. TLR2 priming in BMDMs was performed by aspirating the medium and adding 500 μl/well of culture medium (DMEM + 10% FBS) containing 500 ng/ml Pam3CSK4 for 5h. TLR4 priming of immortalized macrophage was performed by aspirating the medium and adding culture medium (DMEM + 10% FBS) containing 100 ng/ml LPS for 3h. MNV infections were performed using viral dilutions made from a stock solution stored at -80°C in cell culture medium. First, 250 μl of medium was aspirated and then either 250 μl of medium was added (mock-infected controls), 250 μl of UV-inactivated MNV was added, or 250 μl of live MNV was added, the latter two both at a multiplicity of infection (MOI) 5. Control UV-inactivated MNV was prepared by placing the virus under UV light for at least 1h. After the infection the cells were incubated for 1h at 37°C with regular shaking, after which the medium was removed, the cells were washed with PBS and 1 ml culture medium was added. The cells were then incubated for indicated time periods at 37°C and 5% CO_2_ before sample collection. For canonical Nlrp3 inflammasome triggering, BMDMs were stimulated with 1 μg/ml Ultrapure LPS from Salmonella Minnesota (Invivogen) for 3 hours, after which ATP (5 mM, Roche), Nigericin (20 μM, Sigma Aldrich) or Silica (0.5 mg/ml, Min—U—Sil5 US Silica) were applied for 45 min, 45 min and 6 hours, respectively, in 37°C and 5% CO_2_ cell culture conditions.

### Cellular membrane integrity and apoptotic caspase assays

Cellular membrane integrity and Caspase-3/7 enzymatic activity were determined with an IncuCyte FLR imaging system (Essen Bioscience), using the non-cell-permeable SYTOX Green (SG) DNA staining agent (30 nM) (Invitrogen) and the Casp-3/7 fluorogenic substrate (1:46 v/v) (Life technologies), respectively, according to the manufacturer’s protocol. The percentage of positive cells was calculated with the IncuCyte software package. These percentages were normalized to cell confluency as well as to a 100% cell count control achieved by SG-labelling of Triton X-100 treated wells, and were corrected for the number of positive cells observed at time point 0.

### *In vivo* gastrointestinal MNV infections

Age- and sex-matched mice were infected by oral gavage with the MNV-1 CW1 strain. The infectious dose used was 10^6^ or 10^7^ PFU per mouse, as indicated, administered in 100 μl PBS. Survival was monitored daily. Peyer`s patches, mesenteric lymph nodes, spleen, liver, ileum and stool were collected at indicated days post-infection for homogenization and further analysis.

### Cytokine and Lipocalin-2 quantifications

Tissue samples were weighed and were homogenized in 500 μl PBS, after which lysis was completed by addition of lysis buffer (20 mM Tris HCl (pH 7.4), 200 mM NaCl, 1% Nonidet P-40) and incubation for 10 minutes on ice. Full speed centrifugation for 30 minutes cleared the homogenate and supernatant was used for further analysis. Mouse cytokines in cell culture supernatants and tissue homogenates were determined by magnetic bead-based multiplex assay using Luminex technology (Bio-Rad) and type I IFN levels were analyzed using eBioscience IFNα/IFNβ Procartaplex, all according to the manufacturer’s protocols. In S5E Fig, IL-1β was detected by the BD OptEIA ELISA kit (BD biosciences) according to the manufacturer’s protocol. Cytokines from tissue homogenates were normalized to weight of tissue, while cytokines from cell culture supernatants were expressed as concentration per ml of cell culture medium. For measuring Lipocalin-2 levels, fecal pellets were weighed and were homogenized in 500μl PBS using sterile soil grinding SK38 2ml tubes (Bertin Technologies). Stool homogenates were collected in 1.5 ml eppendorf tubes and cleared upon full speed centrifugation for 30 minutes. Lipocalin-2 levels in stool supernatants were then analyzed using the mouse Lipocalin-2/NGAL duoset ELISA (R&D systems) according to the manufacturer’s instructions, and were normalized per mg of stool.

### Quantitative real time PCR

Tissue samples collected on indicated days were homogenized in 500 μl TRIsure (Bioline). For RNA isolation from primary macrophages grown in monolayer, cells were directly lysed in the culture plate by adding 700 μl of TRIsure. Cell lysate was pipetted up and down to ensure sufficient cell disruption. Total RNA isolation was performed according the manufacturer’s protocol. cDNA was synthesized using the iScript gDNA clear cDNA Synthesis kit (Biorad) and quantitative PCR was performed using Taqman gene expression assays (Life technologies). Ifnβ mRNA levels were normalized to the levels of the Tbp reference gene. To determine MNV genome copy numbers, cell culture supernatants were aspirated, after which adherent BMDMs were lysed in TRIsure. RNA was isolated and qRT-PCR was performed using the specific 5’-CCGCAGGAACGCTCAGCAG-3’ and 5’-GGCTGAATGGGGACGGCCTG-3’ primers together with the custom made Taqman probe 5’-ATGAGTGATGGCGCA-3’ [16]. Viral genome copies were quantified according to a serially diluted MNV DNA standard.

### Confocal microscopy

To visualize cell morphology over time, 10^5^ BMDMs per well were seeded onto a poly-L-lysine (Sigma)-coated eight-well slide chamber (ibidi). The next day, the cells were infected with UV-inactivated or live MNV (MOI 5), followed by incubation at 37°C and 5% CO2. After 1 hour of infection, the medium was removed, cells were washed with PBS and 1 ml medium with fluorescent dye was added. To visualize membrane integrity, the non-permeable DNA stain Propidium iodide (BD Bioscience) was added to the medium (50 ng/ml). Cells were imaged every 30 minutes over a time-period of 24 hours on a Zeiss Spinning Disk microscope using a 25x objective.

### Western blotting

Cells and culture supernatants, and tissue homogenates were incubated with cell lysis buffer (20 mM Tris HCl (pH 7.4), 200 mM NaCl, 1% Nonidet P-40) and denatured in Laemlli buffer by boiling for 10 min. Proteins were separated by SDS-PAGE electrophoresis (Thermo Scientific) after which proteins were transferred to nitrocellulose membranes (Thermo Scientific) using semi-dry (20 min) or turbo (7 min) blotting. Blocking and antibody incubation were performed in PBS or TBS supplemented with 0.05% Tween20 (vol/vol) and 3% or 5% (wt/vol) non-fat dry milk. The membranes were incubated overnight at 4°C with primary antibodies against Caspase-1 (1:1000; Adipogen), IL-1β (1:2000; GeneTex), Nlrp3 (1:1000; Adipogen), Caspase-11 (1:1000; Novus biologicals), GSDMD (1:1000)[29], ASC (1:1000; ECM Biosciences), MLKL (1:1000; Millipore), or Caspase-3 (1:1000; Cell signaling). After washing, membranes were incubated with HRP-conjugated anti-mouse, anti-rabbit or anti-rat secondary antibodies (1:5000; Jackson ImmunoResearch Laboratories, 111-035-144, 112-035-143, 112-035-143) or were incubated with the directly labeled primary antibody β-actin-HRP (1:10000; Santa Cruz) for up to 3 h. Proteins of interest were detected by the enhanced SuperSignal West Pico Chemiluminescent Substrate (Thermo Scientific).

### Statistical analysis

For log-linear regression analysis of the cytokine and chemokine expressions shown in Fig 1A, a Generalized Linear Mixed Model (GLMM) (fixed model: poisson distribution, log link; random model: gamma distribution, log link) as implemented in Genstat v18 (VSN International, Hemel Hempstead, UK; www.genstat.co.uk) was fit to the data. The linear predictor vector of the values was written as follows: log(μ) = η = Xβ + Zν, where the matrix X is the design matrix for the fixed terms (i.e. genotype, treatment and tissue) and their interactions, β is their vector of regression coefficients, Z is the design matrix for the random term (i.e. subject), and ν is the corresponding vector of random effect having a gamma distribution. The significance of the regression coefficients was assessed by a t-test. Estimated mean values and their standard errors were obtained as predictions from the GLMM, formed on the scale of the response variable. Other statistics used include two-sided Student’s t-test with unequal variance and nonparametric log-rank test, both analyzed with Prism 7.0 (GraphPad Software, San Diego, CA).

## Supporting information

S1 Fig STAT1 prevents host lethality and viral replication upon gastrointestinal MNV infection. **(A)** Kaplan-Meier survival curve of Stat1^+/-^ (n = 4) and Stat1^-/-^ (n = 7) littermates, infected with 10^7^ PFU live MNV via oral gavage. No further deaths occurred beyond 7 days post-infection. (**B-J)** Age- and sex-matched Stat1^+/-^ and Stat1^-/-^ littermates were infected with 10^7^ PFU live MNV via oral gavage. Indicated organs were collected 1, 2 or 3 days post-infection. qRT-PCR was performed to determine MNV genome copy numbers. Statistics (B-J): Two-sided Student’s t-test with unequal variance; ns not significant; *p<0.05; **p<0.01; ***p<0.001.
(TIF)

S2 Fig Increased MNV loads in Stat1^-/-^ mice are associated with anti-viral type I IFN production. Stat1^+/-^ and Stat1^-/-^ littermates were infected with 10^7^ PFU of live MNV via oral gavage, after which the indicated **(A)** gastrointestinal or **(B)** peripheral tissues were collected at 3 days post-infection. qRT-PCR was performed to determine IFNβ mRNA production. Statistics: Mann Whitney U test; ns not significant; *p<0.05; **p<0.01; ***p<0.001.
(TIF)

S3 Fig Stat1^-/-^ mice display diarrhea and increased fecal Lipocalin-2 levels after gastrointestinal MNV infection. Stat1^+/-^ and Stat1^-/-^ littermates were infected with 10^7^ PFU UV-inactivated or live MNV via oral gavage. Stool was collected at indicated time points and analyzed **(A)** for fecal consistency and **(B)** for Lipocalin-2 levels by ELISA. Data shown are combined from two independent experiments. Statistics: Mann Whitney U-test; ns not significant; **p<0.01, *** p<0.001.
(TIF)

S4 Fig Kinetics of IL-1β production upon gastrointestinal MNV infection. Stat1^+/-^ and Stat1^-/-^ littermates were infected with 10^7^ PFU of UV-inactivated MNV or live MNV via oral gavage. **(A-E)** IL-1β production was determined via Multiplex ELISA in indicated tissues collected at 1, 2 or 3 days post-infection, and was plotted relative to the weight of the collected tissue sample. Statistics: Log-linear regression analysis, p values indicate association with Stat1^-/—^Live-MNV set-up, with **p<0.01; ***p<0.001.
(TIF)

S5 Fig MNV specifically activates the Nlrp3 inflammasome in primary as well as immortalized murine macrophages. Bone-marrow-derived macrophages (BMDMs) from **(A-B)** WT, Nlrp3^-/-^, Nlrc4^-/-^ and Aim2^-/-^ mice; or from **(C-D)** WT, Nlrp3^-/-^ and Nlrp6^-/-^ mice were left untreated (unprimed) or TLR2-primed for 5 hours, after which they were infected with either UV-inactivated or live MNV, both at a MOI 5. 24 hours post-infection culture supernatant was analyzed for **(A, C)** secreted IL-1β and **(B, D)** cell lysates were immunoblotted for IL-1β and caspase-1 maturation. **(E)** WT, Caspase-1^-/-^, Asc^-/-^ and Nlrp3^-/-^ immortalized BMDMs were left unprimed or were pre-treated with 100ng/ml lipopolysaccharide (TLR4-primed) for 3h followed by MNV infection (MOI 5). Supernatants were collected 16 hours post-infection and IL-1β was measured by ELISA. Data in **(A, C)** are the means ± SD from a single experiment using triplicate wells. Data in **(B, D)** are from a single experiment. Data in **(E)** are means ± SEM of triplicate wells; experiment shown is representative of 3 independent experiments.
(TIF)

S6 Fig Abrogating Nlrp3 inflammasome activation does not impair MNV replication or anti-viral IFNβ production in macrophages. Bone-marrow-derived macrophages (BMDMs) from WT or Nlrp3^-/-^ mice were left untreated (unprimed) or TLR2-primed for 5 hours, after which they were infected with either UV-inactivated or live MNV, both at a MOI 5. **(A)** qRT-PCR was performed from infected BMDMs at indicated time points to determine MNV genome copies. **(B)** Culture supernatant was analyzed 24 hours post-infection for secreted IFNβ by ELISA. Data shown represent the means ± SD of BMDMs derived from 3 mice per genotype, each performed in triplicate.
(TIF)

S7 Fig MLKL is not involved in MNV-induced membrane permeability, inflammasome activation or IL-1β secretion. Bone-marrow-derived macrophages (BMDMs) from Mlkl^+/-^ or Mlkl^-/-^ littermates were left untreated (unprimed) or TLR2-primed for 5 hours and were then infected with MNV. **(A, B)** Incucyte real-time analysis of Sytox Green (SG) uptake in MNV-infected **(A)** unprimed and **(B)** TLR2-primed BMDMs for 24 hours, monitoring every 30 minutes. **(C)** Culture supernatant from TLR2-primed BMDMs infected with MNV for indicated time periods were analyzed for secreted IL-1β by ELISA. **(D, E)** Cell lysates of MNV-infected **(D)** TLR2-primed or **(E)** unprimed BMDMs were collected at indicated time periods and immunoblotted for IL-1β and caspase-1 maturation. Data shown in **(A, B)** are the means of duplicate wells from a representative experiment out of two independent experiments; data in **(C)** are the means ± SD of triplicate wells from a representative experiment out of two independent experiments. Data in **(D, E)** are representative of two independent experiments.
(TIF)

S8 Fig Gsdmd^-/-^ macrophages display increased caspase-3 and -7 enzymatic activity upon MNV infection. Bone-marrow-derived macrophages (BMDMs) from WT and Gsdmd^-/-^ mice were left unprimed, after which they were infected with live MNV at MOI 5. **(A)** Casp3/7 enzymatic activity was assessed for 24 hours by IncuCyte real-time monitoring every 30 minutes. **(B)** Cell lysates were collected at indicated time points and immunoblotted for Caspase-3 cleavage. Data shown in **(A)** are the means of duplicate wells from a representative experiment out of two independent experiments. Data shown in **(B)** is representative of three independent experiments.
(TIF)

S9 Fig STAT1 deficiency does not affect canonical Nlrp3 inflammasome activation by non-viral triggers. **(A-C)** Bone-marrow-derived macrophages (BMDM) from Stat1^+/-^ and Stat1^-/-^ mice were treated with LPS alone (lanes 1 and 4), with **(A)** ATP, **(B)** Nigericin or **(C)** MSU alone (lanes 2 and 5), or were pre-treated with LPS for 3 hours before **(A)** ATP, **(B)** Nigericin or **(C)** MSU was applied (lanes 3 and 6). **(A-B)** 45 minutes or **(C)** six hours after stimulation cell lysates were collected and immunoblotted for IL-1β. Data shown are representative of two independent experiments.
(TIF)

S10 Fig MAVS deficiency abrogates MNV-induced type I IFN production but does not affect MNV-induced inflammasome activation in macrophages. **(A-B)** Bone-marrow-derived macrophages (BMDM) from WT and Mavs^-/-^ mice were left untreated (unprimed) or were TLR2-primed for 5 hours as indicated, before infection with UV-inactivated or live MNV at MOI 5. At 24 hours post-infection cell culture supernatant was analyzed for secreted **(A)** IFNβ and **(B)** IL-1β levels by ELISA; and **(C-D)** cell lysates were immunoblotted for IL-1β, caspase-1, GSDMD and caspase-3 cleavage. **(E)** At indicated times post-infection RNA was isolated from BMDMs to determine MNV genome copy numbers. Data shown in **(A, B)** are the means ± SD of triplicate wells from a representative experiment out of 2 independent experiments. Data shown in **(C, D)** are representative for 2 independent experiments. Data shown in **(E)** represent means ± SD of BMDMs derived from three mice per genotype, infected in three independent experiments each with triplicate wells.
(TIF)

S11 Fig Stat1^-/-^Mavs^-/-^ macrophages do not produce type I IFN in response to MNV infection. Unprimed bone-marrow-derived macrophages (BMDM) from Stat1^+/-^, Stat1^-/-^ and Stat1^-/-^Mavs^-/-^ mice were mock infected (control) or infected with live MNV at MOI 5. After 24 hours supernatant was collected for analysis of secreted IFNβ levels via ELISA. Data shown in are the means ± SD of triplicate wells from a representative experiment out of 2 independent experiments.
(TIF)

S1 Movie Confocal spinning disk microscopy of unprimed WT BMDMs infected with live MNV at MOI2 and provided with Propidium Iodide in the culture medium. Time-lapse images were collected at 1-hour intervals over 24 hours. Representative of at least 2 independent experiments.
(MP4)

S1 Table Raw values of multiplex ELISA from Fig 1A with statistical analyses. Age- and sex-matched Stat1^+/-^ and Stat1^-/-^ littermates were infected with 10^7^ PFU of UV-inactivated (UV) or live MNV via oral gavage. At 3 days post-infection ileum, Peyer`s patches (PP), mesenteric lymph nodes (MLN), spleen and liver lysates were prepared for Multiplex ELISA. Statistics: Log-linear regression analysis, p values indicate association with Stat1^-/-^Live-MNV set-up.
(XLS)
