# Supplementary figures and images for: Nlrp3 inflammasome activation and Gasdermin D-driven pyroptosis are immunopathogenic upon gastrointestinal norovirus infection
Source: PLoS Pathog. 2019 Apr 24;15(4):e1007709. doi: 10.1371/journal.ppat.1007709 (PMC6502405; doi:10.1371/journal.ppat.1007709)

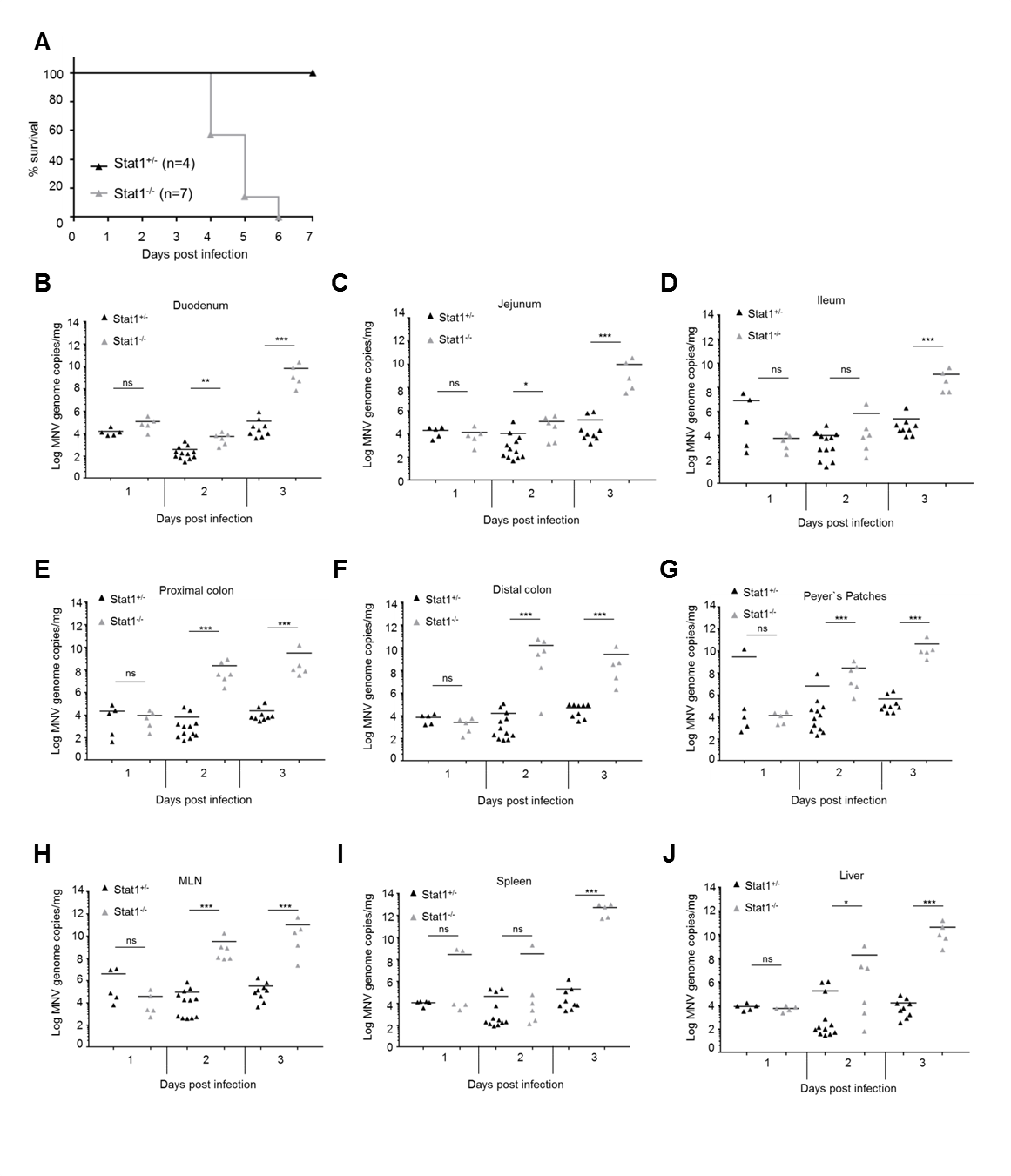

Supplement: S1 Fig — (A) Kaplan-Meier survival curve of Stat1+/- (n = 4) and Stat1-/- (n = 7) littermates, infected with 107 PFU live MNV via oral gavage. No further deaths occurred beyond 7 days post-infection. (B-J) Age- and sex-matched Stat1+/- and Stat1-/- littermates were infected with 107 PFU live MNV via oral gavage. Indicated organs were collected 1, 2 or 3 days post-infection. qRT-PCR was performed to determine MNV genome copy numbers. Statistics (B-J): Two-sided Student’s t-test with unequal variance; ns not significant; *p<0.05; **p<0.01; ***p<0.001. (TIF) [file ppat.1007709.s001.tif]

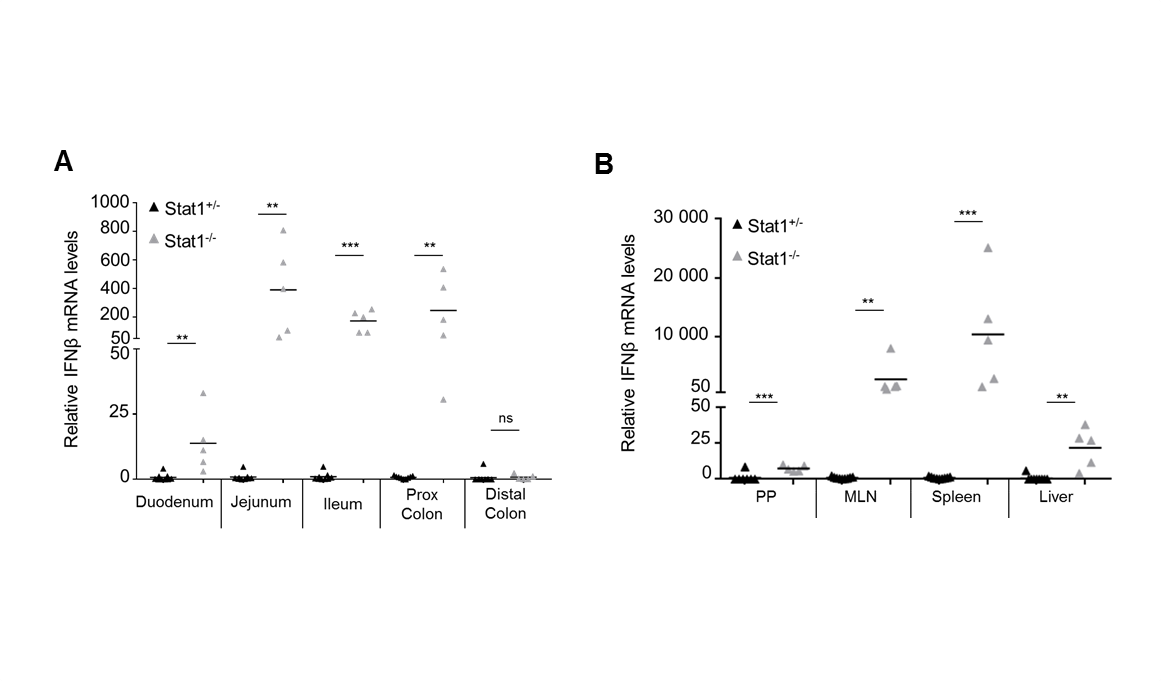

Supplement: S2 Fig — Stat1+/- and Stat1-/- littermates were infected with 107 PFU of live MNV via oral gavage, after which the indicated (A) gastrointestinal or (B) peripheral tissues were collected at 3 days post-infection. qRT-PCR was performed to determine IFNβ mRNA production. Statistics: Mann Whitney U test; ns not significant; *p<0.05; **p<0.01; ***p<0.001. (TIF) [file ppat.1007709.s002.tif]

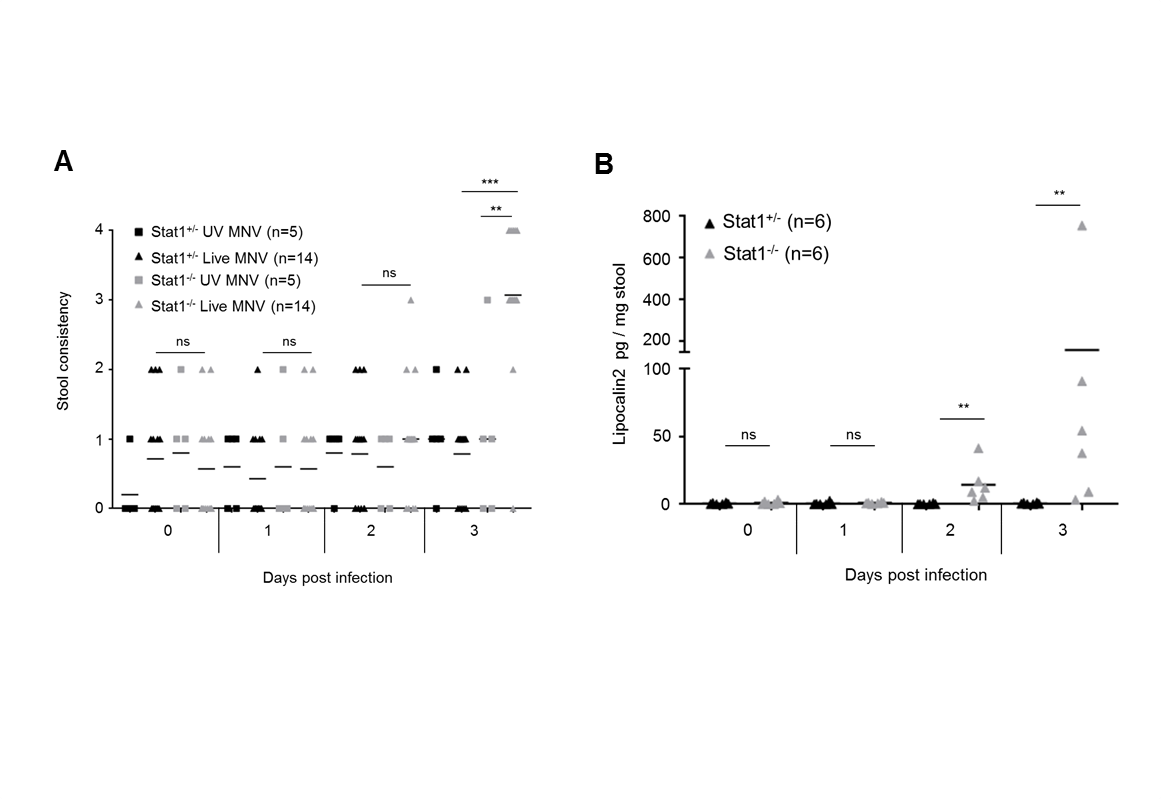

Supplement: S3 Fig — Stat1+/- and Stat1-/- littermates were infected with 107 PFU UV-inactivated or live MNV via oral gavage. Stool was collected at indicated time points and analyzed (A) for fecal consistency and (B) for Lipocalin-2 levels by ELISA. Data shown are combined from two independent experiments. Statistics: Mann Whitney U-test; ns not significant; **p<0.01, *** p<0.001. (TIF) [file ppat.1007709.s003.tif]

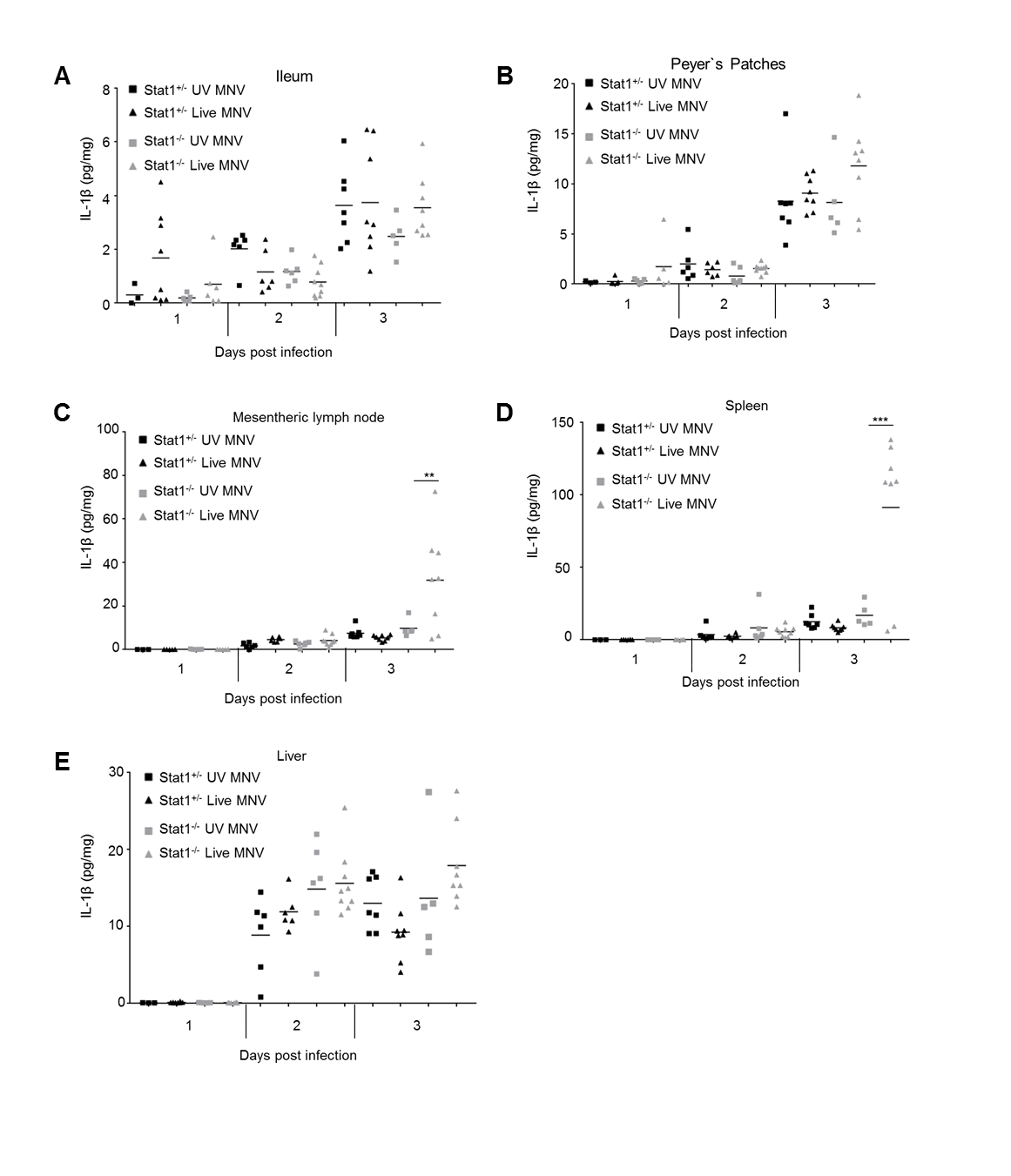

Supplement: S4 Fig — Stat1+/- and Stat1-/- littermates were infected with 107 PFU of UV-inactivated MNV or live MNV via oral gavage. (A-E) IL-1β production was determined via Multiplex ELISA in indicated tissues collected at 1, 2 or 3 days post-infection, and was plotted relative to the weight of the collected tissue sample. Statistics: Log-linear regression analysis, p values indicate association with Stat1-/—Live-MNV set-up, with **p<0.01; ***p<0.001. (TIF) [file ppat.1007709.s004.tif]

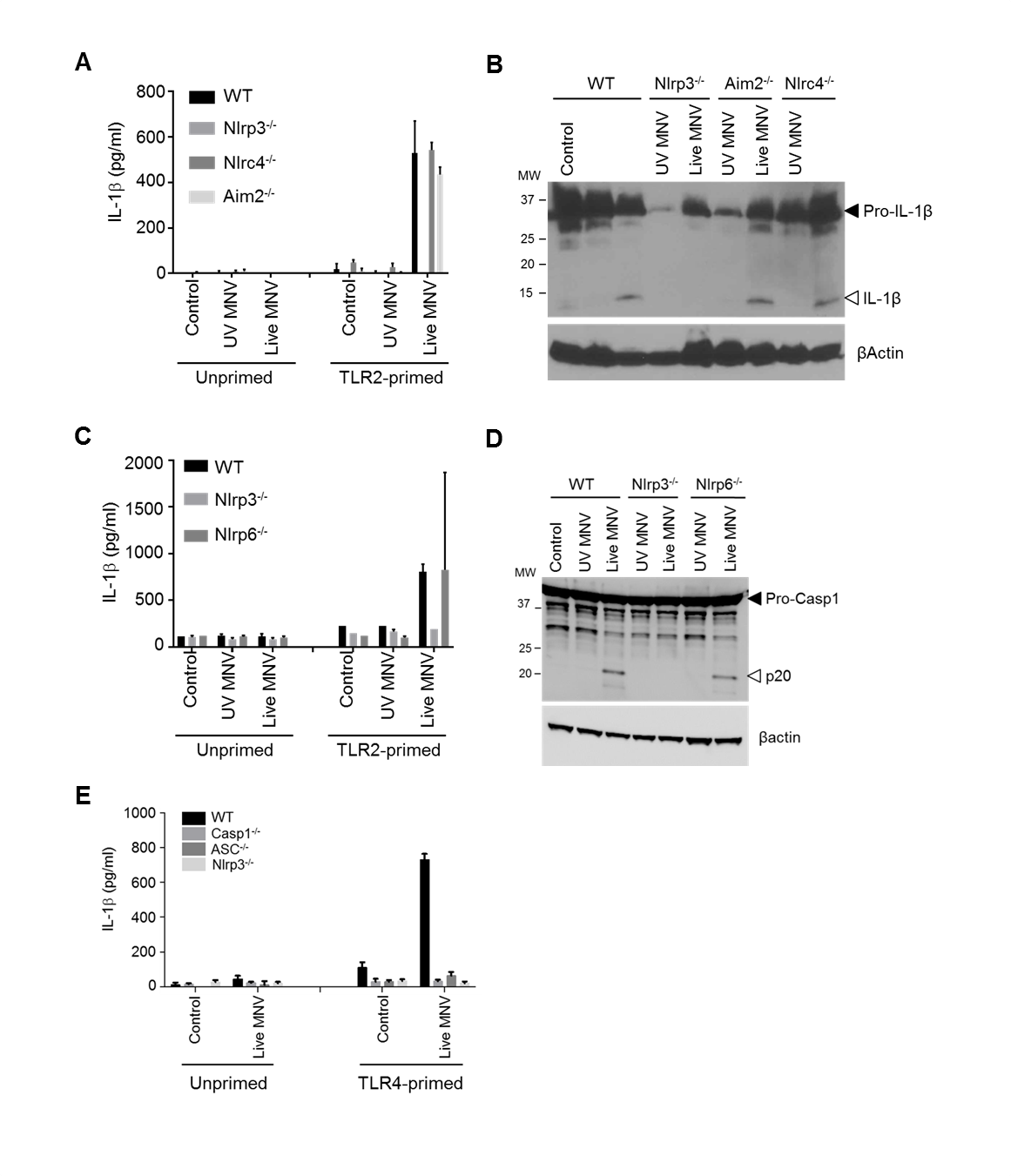

Supplement: S5 Fig — Bone-marrow-derived macrophages (BMDMs) from (A-B) WT, Nlrp3-/-, Nlrc4-/- and Aim2-/- mice; or from (C-D) WT, Nlrp3-/- and Nlrp6-/- mice were left untreated (unprimed) or TLR2-primed for 5 hours, after which they were infected with either UV-inactivated or live MNV, both at a MOI 5. 24 hours post-infection culture supernatant was analyzed for (A, C) secreted IL-1β and (B, D) cell lysates were immunoblotted for IL-1β and caspase-1 maturation. (E) WT, Caspase-1-/-, Asc-/- and Nlrp3-/- immortalized BMDMs were left unprimed or were pre-treated with 100ng/ml lipopolysaccharide (TLR4-primed) for 3h followed by MNV infection (MOI 5). Supernatants were collected 16 hours post-infection and IL-1β was measured by ELISA. Data in (A, C) are the means ± SD from a single experiment using triplicate wells. Data in (B, D) are from a single experiment. Data in (E) are means ± SEM of triplicate wells; experiment shown is representative of 3 independent experiments. (TIF) [file ppat.1007709.s005.tif]

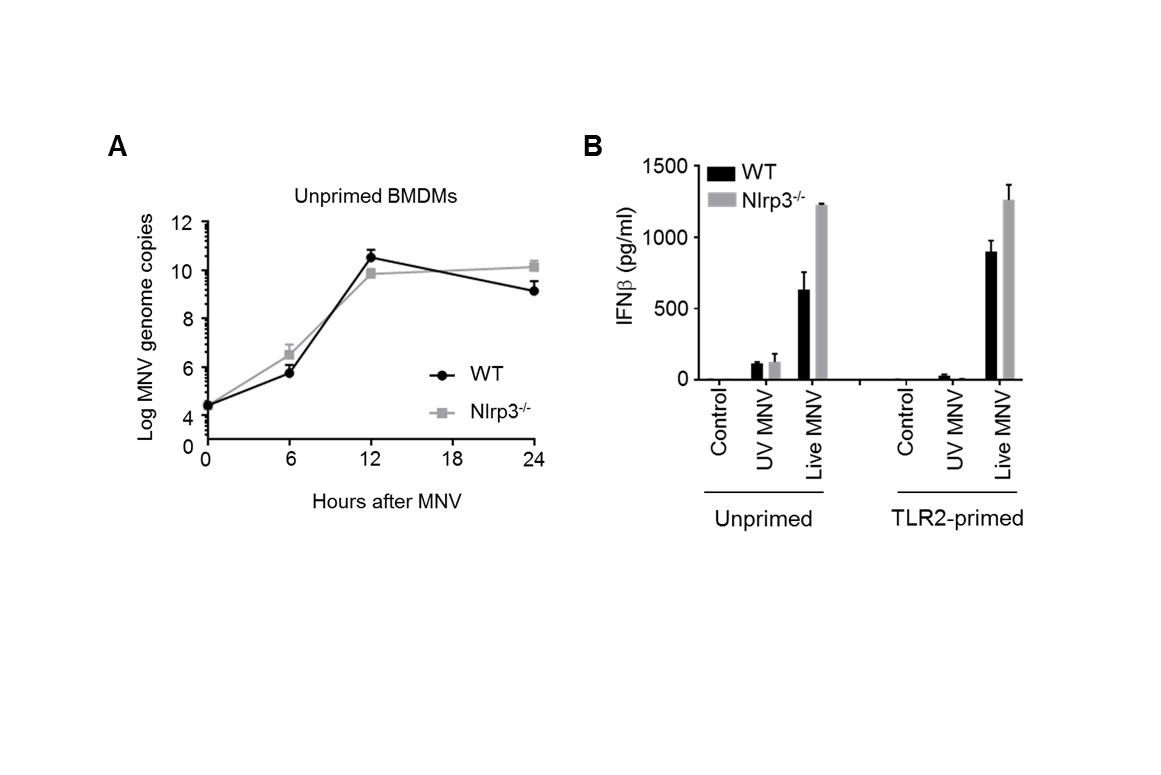

Supplement: S6 Fig — Bone-marrow-derived macrophages (BMDMs) from WT or Nlrp3-/- mice were left untreated (unprimed) or TLR2-primed for 5 hours, after which they were infected with either UV-inactivated or live MNV, both at a MOI 5. (A) qRT-PCR was performed from infected BMDMs at indicated time points to determine MNV genome copies. (B) Culture supernatant was analyzed 24 hours post-infection for secreted IFNβ by ELISA. Data shown represent the means ± SD of BMDMs derived from 3 mice per genotype, each performed in triplicate. (TIF) [file ppat.1007709.s006.tif]

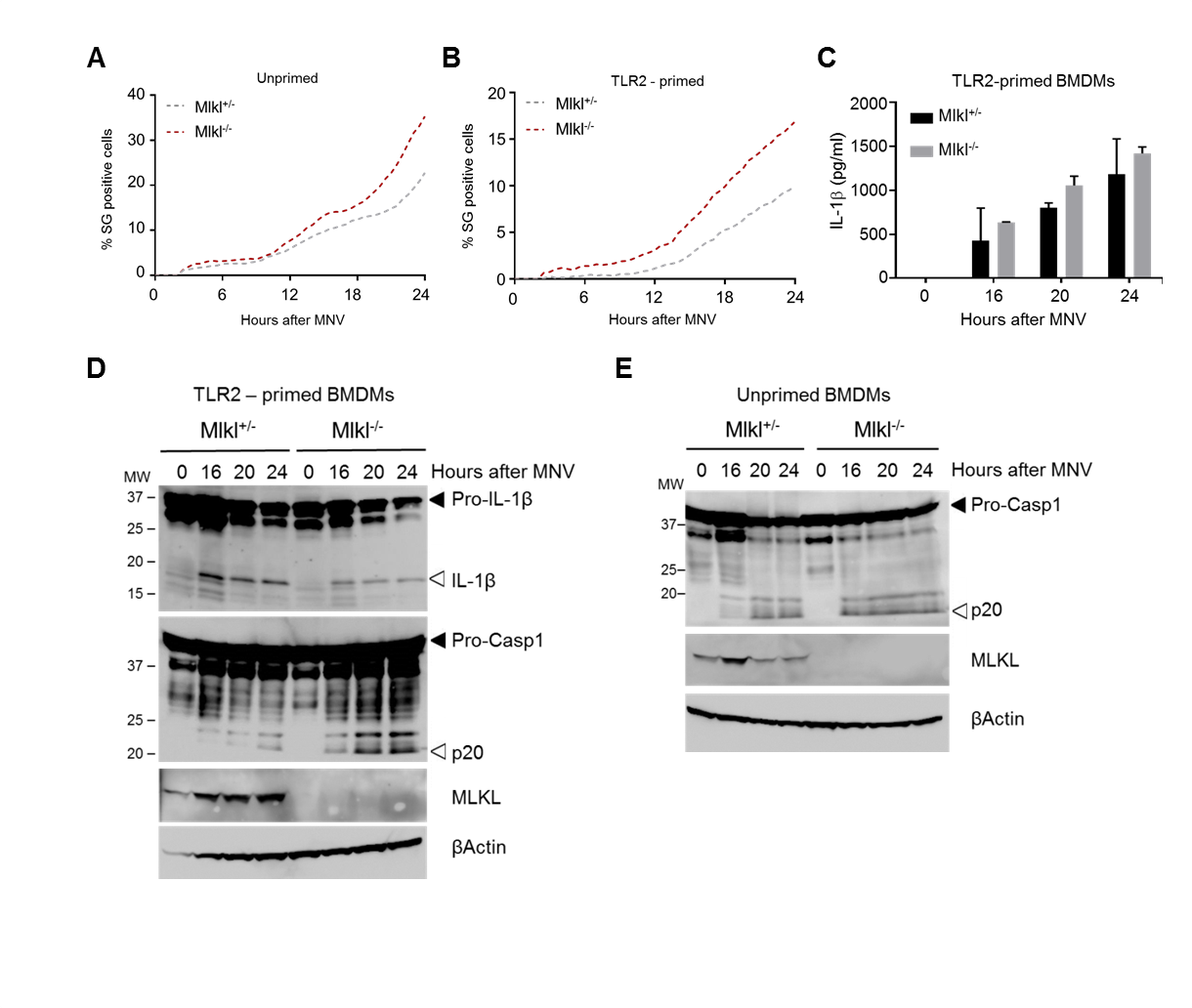

Supplement: S7 Fig — Bone-marrow-derived macrophages (BMDMs) from Mlkl+/- or Mlkl-/- littermates were left untreated (unprimed) or TLR2-primed for 5 hours and were then infected with MNV. (A, B) Incucyte real-time analysis of Sytox Green (SG) uptake in MNV-infected (A) unprimed and (B) TLR2-primed BMDMs for 24 hours, monitoring every 30 minutes. (C) Culture supernatant from TLR2-primed BMDMs infected with MNV for indicated time periods were analyzed for secreted IL-1β by ELISA. (D, E) Cell lysates of MNV-infected (D) TLR2-primed or (E) unprimed BMDMs were collected at indicated time periods and immunoblotted for IL-1β and caspase-1 maturation. Data shown in (A, B) are the means of duplicate wells from a representative experiment out of two independent experiments; data in (C) are the means ± SD of triplicate wells from a representative experiment out of two independent experiments. Data in (D, E) are representative of two independent experiments. (TIF) [file ppat.1007709.s007.tif]

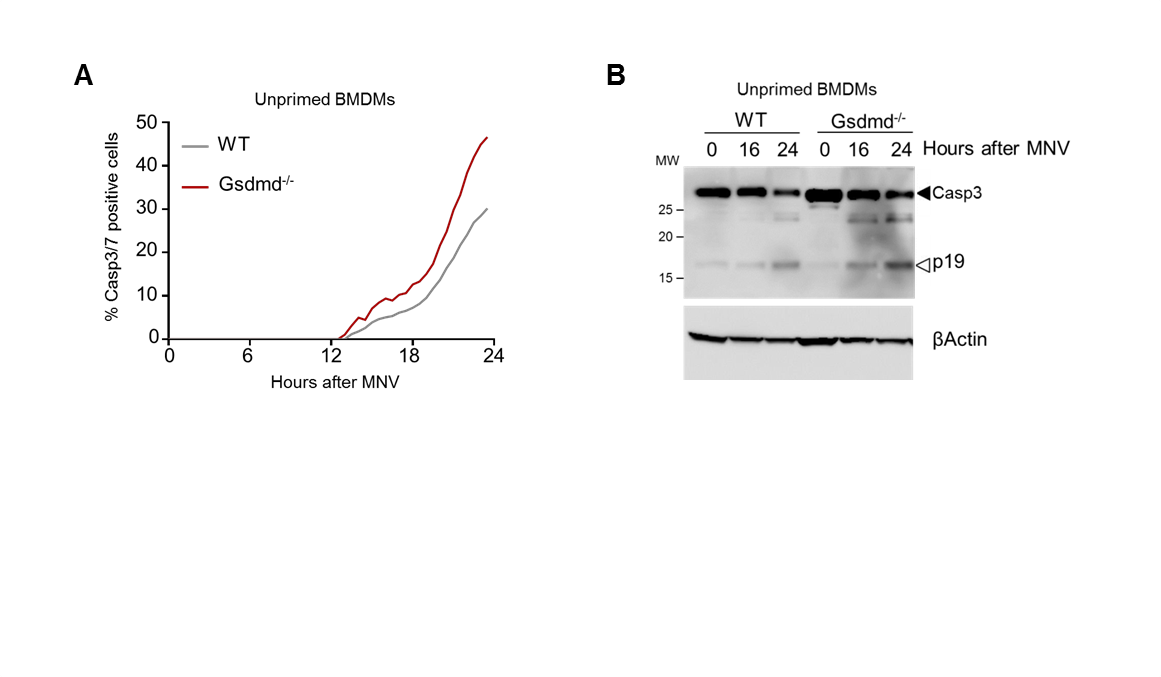

Supplement: S8 Fig — Bone-marrow-derived macrophages (BMDMs) from WT and Gsdmd-/- mice were left unprimed, after which they were infected with live MNV at MOI 5. (A) Casp3/7 enzymatic activity was assessed for 24 hours by IncuCyte real-time monitoring every 30 minutes. (B) Cell lysates were collected at indicated time points and immunoblotted for Caspase-3 cleavage. Data shown in (A) are the means of duplicate wells from a representative experiment out of two independent experiments. Data shown in (B) is representative of three independent experiments. (TIF) [file ppat.1007709.s008.tif]

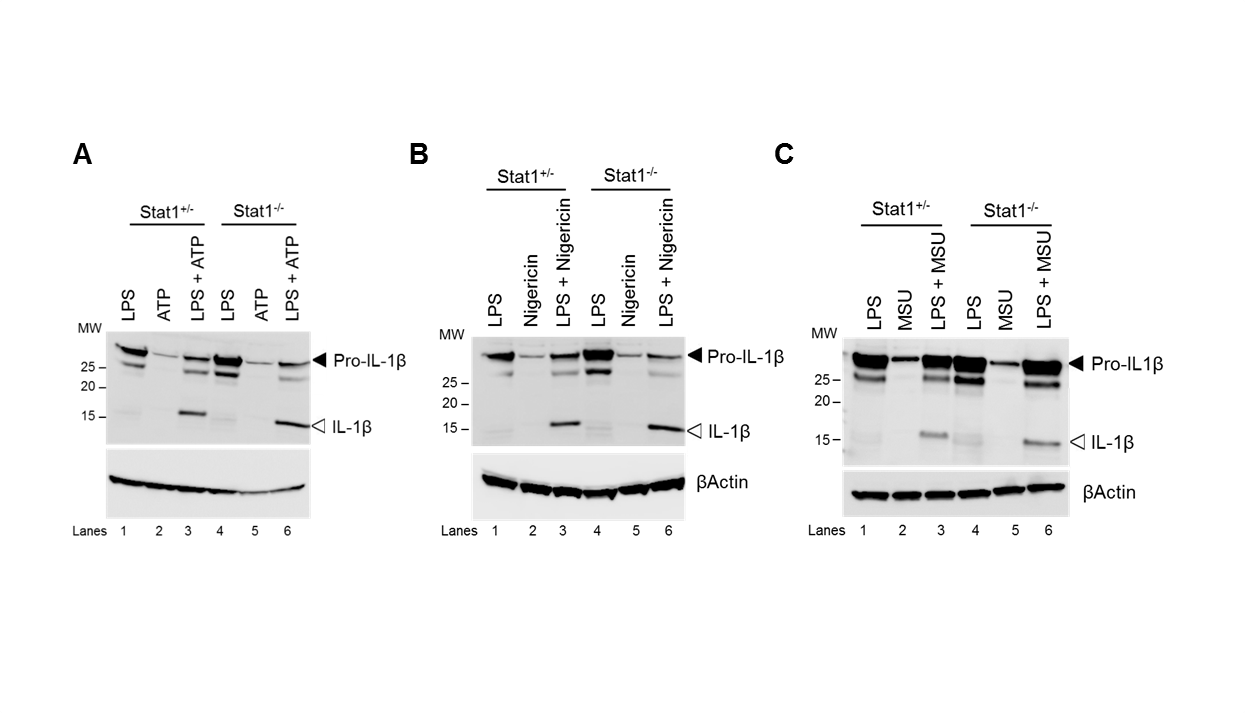

Supplement: S9 Fig — (A-C) Bone-marrow-derived macrophages (BMDM) from Stat1+/- and Stat1-/- mice were treated with LPS alone (lanes 1 and 4), with (A) ATP, (B) Nigericin or (C) MSU alone (lanes 2 and 5), or were pre-treated with LPS for 3 hours before (A) ATP, (B) Nigericin or (C) MSU was applied (lanes 3 and 6). (A-B) 45 minutes or (C) six hours after stimulation cell lysates were collected and immunoblotted for IL-1β. Data shown are representative of two independent experiments. (TIF) [file ppat.1007709.s009.tif]

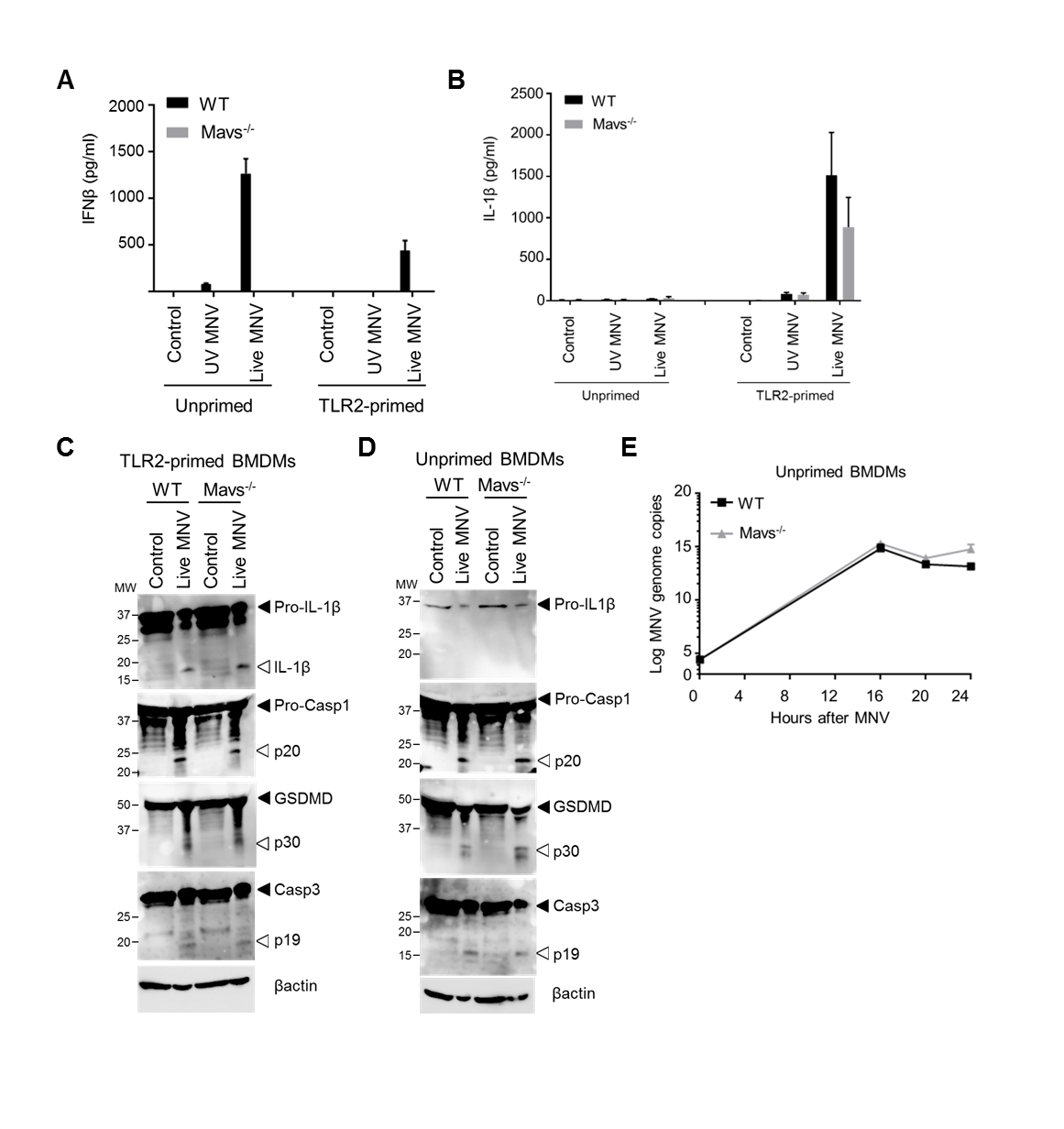

Supplement: S10 Fig — (A-B) Bone-marrow-derived macrophages (BMDM) from WT and Mavs-/- mice were left untreated (unprimed) or were TLR2-primed for 5 hours as indicated, before infection with UV-inactivated or live MNV at MOI 5. At 24 hours post-infection cell culture supernatant was analyzed for secreted (A) IFNβ and (B) IL-1β levels by ELISA; and (C-D) cell lysates were immunoblotted for IL-1β, caspase-1, GSDMD and caspase-3 cleavage. (E) At indicated times post-infection RNA was isolated from BMDMs to determine MNV genome copy numbers. Data shown in (A, B) are the means ± SD of triplicate wells from a representative experiment out of 2 independent experiments. Data shown in (C, D) are representative for 2 independent experiments. Data shown in (E) represent means ± SD of BMDMs derived from three mice per genotype, infected in three independent experiments each with triplicate wells. (TIF) [file ppat.1007709.s010.tif]

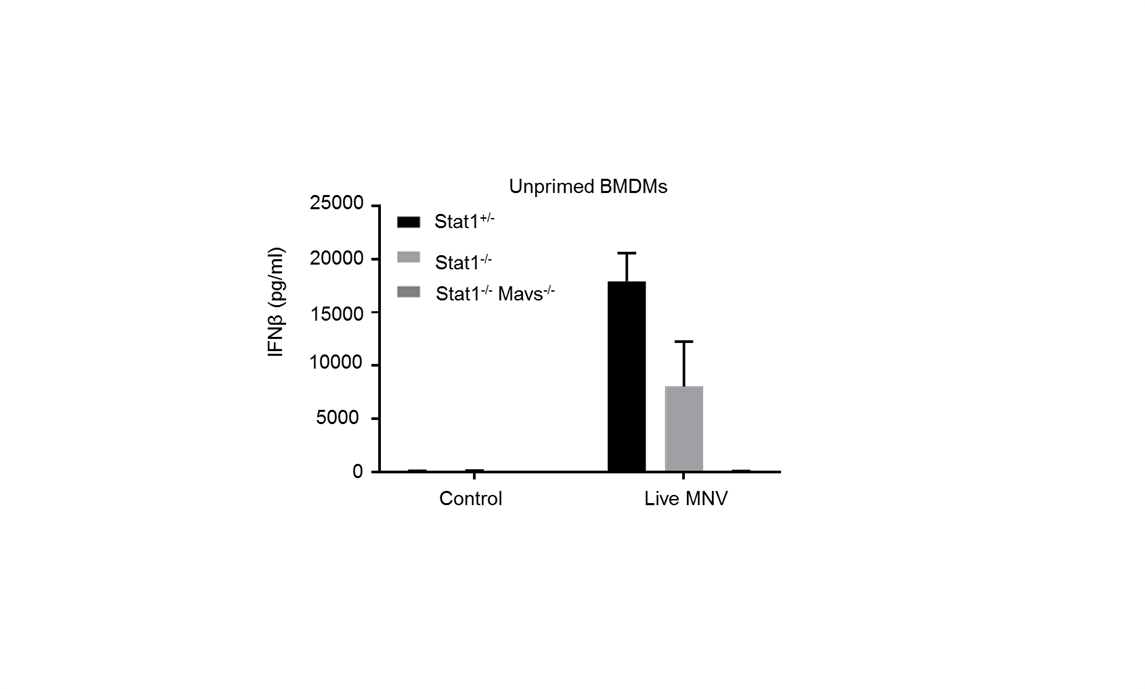

Supplement: S11 Fig — Unprimed bone-marrow-derived macrophages (BMDM) from Stat1+/-, Stat1-/- and Stat1-/-Mavs-/- mice were mock infected (control) or infected with live MNV at MOI 5. After 24 hours supernatant was collected for analysis of secreted IFNβ levels via ELISA. Data shown in are the means ± SD of triplicate wells from a representative experiment out of 2 independent experiments. (TIF) [file ppat.1007709.s011.tif]
